# Valproic acid exposure decreases neurogenic potential of outer radial glia in human brain organoids

**DOI:** 10.3389/fnmol.2022.1023765

**Published:** 2022-11-29

**Authors:** Zhenle Zang, Huachun Yin, Zhulin Du, Ruxin Xie, Ling Yang, Yun Cai, Liuyongwei Wang, Dandan Zhang, Xin Li, Tianyao Liu, Hong Gong, Junwei Gao, Hui Yang, Margaret Warner, Jan-Ake Gustafsson, Haiwei Xu, Xiaotang Fan

**Affiliations:** ^1^Department of Developmental Neuropsychology, School of Psychology, Third Military Medical University (Army Medical University), Chongqing, China; ^2^Department of Neurosurgery, Xinqiao Hospital, Third Military Medical University (Army Medical University), Chongqing, China; ^3^Center for Nuclear Receptors and Cell Signaling, University of Houston, Houston, TX, United States; ^4^Department of Biosciences and Nutrition, Karolinska Institute, Huddinge, Sweden; ^5^Southwest Hospital and Southwest Eye Hospital, Third Military Medical University (Army Medical University), Chongqing, China

**Keywords:** valproic acid, dorsal forebrain organoid, neurogenesis, autism spectrum disorder, outer radial glia

## Abstract

Valproic acid (VPA) exposure during pregnancy leads to a higher risk of autism spectrum disorder (ASD) susceptibility in offspring. Human dorsal forebrain organoids were used to recapitulate course of cortical neurogenesis in the developing human brain. Combining morphological characterization with massive parallel RNA sequencing (RNA-seq) on organoids to analyze the pathogenic effects caused by VPA exposure and critical signaling pathway. We found that VPA exposure in organoids caused a reduction in the size and impairment in the proliferation and expansion of neural progenitor cells (NPCs) in a dose-dependent manner. VPA exposure typically decreased the production of outer radial glia-like cells (oRGs), a subtype of NPCs contributing to mammalian neocortical expansion and delayed their fate toward upper-layer neurons. Transcriptomics analysis revealed that VPA exposure influenced ASD risk gene expression in organoids, which markedly overlapped with irregulated genes in brains or organoids originating from ASD patients. We also identified that VPA-mediated Wnt/β-catenin signaling pathway activation is essential for sustaining cortical neurogenesis and oRGs output. Taken together, our study establishes the use of dorsal forebrain organoids as an effective platform for modeling VPA-induced teratogenic pathways involved in the cortical neurogenesis and oRGs output, which might contribute to ASD pathogenesis in the developing brain.

## Introduction

Autism spectrum disorder (ASD) is a neurodevelopmental disorder features social and communication deficits and increased stereotypic patterns of behavior (Lordbagh et al., 2018). ASD affects ~1–2% of the population and represents a major public health concern (Lyall et al., 2017). The etiology of ASD likely involves genetic and environmental risk factors and their interaction (Sandin et al., 2014). Emerging evidence has suggested that brain areas essential for social and execution in individuals with ASD are structurally and functionally impaired (Wegiel et al., 2014). In recent decades, growing evidence has led to the concept that abnormal embryonic development in the cerebral cortex, is a common pathophysiological substrate in the development of ASD (Willsey et al., 2013; Trevino et al., 2021).

Rodent models based on modified genes related to ASD and environmental risk factors such as sodium valproate (VPA) exposure recapitulated certain defects in humans with ASD and facilitated dissection of the pathogenesis of ASD (Currais et al., 2016; Sungur et al., 2016; Selimbeyoglu et al., 2017). However, strategies based on animal models remained challenging in the translation of preclinical findings to the clinic due to the disparities in behavior and brain structure between humans and rodents (Hill and Walsh, 2005; Kaiser and Feng, 2015; Hodge et al., 2019; Pembroke et al., 2021). Ethical and practical issues pose a unique challenge to investigating the early brain development of humans and hinder our comprehensive understanding of the pathogenesis of ASD. Recent advanced technologies for culturing cerebral organoids have opened new avenues to investigate human neurodevelopmental mechanisms and disorders (Lancaster et al., 2013; Lancaster and Knoblich, 2014; Kelley and Pașca, 2022). Generation of cortical from human induced pluripotent stem cells (hiPSCs) or human embryonic stem cells (hESCs) have demonstrated a striking resemblance of essential architectural and functional features of the fetal brain at early- and mid-gestation (Qian et al., 2016; Bershteyn et al., 2017).

Two principal NPCs are present in the developing neocortex, referred to as radial glial cells (RGs). The cell bodies of apical RGs (aRGs) located on the apical side of the developing ventricular zone (VZ) generate basal RGs such as intermediate progenitor cells (IPCs) and outer radial glia cells (oRGs) in the subventricular zone (SVZ; Florio and Huttner, 2014). oRGs are abundant in the human cerebral cortex but rare in rodent cortex, and involved in cortex expansion (Reillo et al., 2011; Wang et al., 2011b). More importantly, cortex-like structures formed in cultured cerebral organoids recapitulated human cortical development by producing an outer subventricular zone (oSVZ) -like progenitor layer encompassing oRG-like cells (Shi et al., 2012; Kadoshima et al., 2013; Lancaster et al., 2013; Qian et al., 2016). Indeed, cortical organoids have been applied to ASD model for clarifying phenotypes and mechanisms that cannot be recapitulated utilizing animal models (Mariani et al., 2015; de Jong et al., 2021).

VPA is a highly effective anti-epileptic drug for the treatment of some patients with bipolar disorder and epilepsy. Epidemiological studies in humans have shown that its use during pregnancy was teratogenic to brain development and increased the risk of ASD in offspring (Rasalam et al., 2005; Christensen et al., 2013). Importantly, VPA is still being used during pregnancy, in some patients for whom there is no alternative. Therefore, better interpreting the pathological role of VPA exposure during brain development is critical for uncovering the pathogenesis mechanisms of ASD induced by VPA. Typically, defects in neurogenesis in the prefrontal cortex cause autistic behavior and have been confirmed in primate maternal exposure to VPA (Zhao et al., 2019). Meanwhile, the administration of VPA on gestational day 12.5 in rodents recapitulated certain deformities shown in human fetal exposure to VPA in humans (Kataoka et al., 2013). Strikingly, prenatal VPA exposure has been reported to delay cortical development and inhibit neurogenesis, which contributes to the subsequent development of ASD (Kataoka et al., 2013; Zhao et al., 2019). However, dynamic developmental processes of the cerebral cortex and changes in the architecture of the human brain involved in the ASD pathogenesis induced by prenatal VPA exposure is largely unclear.

In this study, we have explored the effect of VPA exposure on early to middle corticogenesis through chronic activity in hESC-derived dorsal forebrain organoids. We found higher doses of VPA inhibited the expansion in human dorsal forebrain organoids, leading to impairments in the proliferation and expansion of NPCs as well as lamination, which well recapitulated early cortical phenotypes in the pathogenesis of ASD. By combining morphological characterization with massive parallel RNA sequencing (RNA-seq) on organoids, we uncover that VPA exposure in organoids influenced not only the activity of Wnt/β-catenin signaling but also the ASD-related gene expression. Our results further found that early developmental exposure to VPA leads to aberrations in the niche of oSVZ and oRG output in cultured cerebral organoids involved in the pathogenesis of ASD.

## Materials and methods

### hESC culturing and generation of cerebral organoid

hESC line (H9) was obtained from Stem Cell Bank, Chinese Academy of Sciences. After being confirmed without contamination, cells were cultured on the Matrigel-coated 6-well plates in the mTeSR1 medium (05850, Stem Cell Technologies, CA) without feeders. Cerebral organoids were produced according to the Lancaster method (Lancaster and Knoblich, 2014) with slight modifications (Bagley et al., 2017). Briefly, H9 hESCs were digested into a single cell with TrypLE (12,605,028, Gibco, United States). ~9,000 cells were seeded in each well of 96-well plate (CLS7007, Corning, United States) to generate embryoid bodies (EBs) in low-bFGF (4 ng/ml, 100-18B, Peprotech, United States) media with Rock inhibitor Y-27632 (10 μM, SCM075, Millipore, United States) at day 0. EBs were formed and cultured in the 96-well plate for 6 days. On day 6, EBs were transferred to N2-based neural induction media subsequently, in which the smoothened receptor inhibitor cyclopamine A (CycA, 239,803, Calbiochem, Germany) was added to enhance dorsal forebrain identity. On day 12, the EBs were embedded in Matrigel (356,234, BD Biosciences, United States) and were grown in differentiation media with B27 without vitamin A (12,587,010, Invitrogen, United States) for 4 days. On day 16, cerebral organoids were cultured in differentiation medium containing vitamin A utilizing an orbital shaker (17,504,044, Invitrogen, United States). The medium was replaced twice a week.

Brain organoids were treated with VPA (P4543, Sigma, United States, dissolved in distilled water) at three concentrations (100, 250, and 500 μM) from day 16, and distilled water (0 μM) used as a control. Medium containing VPA was changed during the treatment period. For the Valpromide (VPD, V3640, Sigma, United States) or β-catenin antagonist treatment, 1 mM VPD or 10 μM IWR-1 (HY-12238, MedChemExpress, United States) was added to the medium from day 16, and brain organoids were collected at day 28.

### Immunofluorescence

Cerebral organoids were fixed with 4% paraformaldehyde (PFA) for half an hour and rinsed in PBS for three times, then subjected to dehydration with 30% sucrose solution. The dehydrated organoids were frozen in Optimal Cutting Temperature (OCT) compound (VWR) and followed by sectioning (20 μm thick) using Leica CM3050 S Research Cryostat (Leica, Germany). For immunostaining, sections were permeabilized and blocked at room temperature, then were incubated with primary antibodies overnight at 4°C. The antibodies and their dilutions are shown in Supplementary Table S1. Sections were washed with PBS, then treated with fluorescently labeled secondary antibodies (Alexa488 anti-mouse, A-21202, Alexa488 anti-rabbit, A-21206, 1:500, Alexa594 anti-rat, A-21209, Alexa 647 anti-mouse, A-31571, 1:500, Invitrogen, United States; Cy3-conjugated anti-rabbit, 711–165-152 and Cy3-conjugated anti-mouse, 715–165-150, Jackson, United States) for 2 h and washed with PBS for three times. The fluorescent signals were examined by either a Zeiss Axivert microscope (Zeiss, Imager A2, Oberkochen, Germany) or a confocal laser scanning microscope (Zeiss, LSM800; Oberkochen, Germany). Analysis was performed using ImageJ software (National Institute of health, United States).

### EdU Click-iT assay

For EdU labeling, EdU (final concentration 10 μM) was added to the culture media and treated for 2 h, then washed with PBS three times. EdU Click-iT assay was performed on fixed tissue sections as manufacturer’s instruction (C10337, Invitrogen, Thermo Fisher Scientific, United States), followed by immunostaining.

### TUNEL assay

The apoptosis in the forebrain organoids was detected with the TUNEL assay kit (*In Situ* Cell Death Detection Kit, Roche, Switzerland). Briefly, buffers 1 and 2 were mixed at a ratio of 1:9 and diluted the mixture with PBS following instructions and sections were then transferred into the TUNEL reaction mixture at 37°C for 30 min in a humidified dark, followed by immuno-staining.

### Quantification of layer thickness

The thickness of VZ and cortical plate (CP) of the forebrain organoids were measured following the literature (Qian et al., 2016). Briefly, the VZ was determined by SOX2 staining and neural tube shape, the CP was outlined by the region outside the VZ to the nearest pial surface. The length for VZ and CP layer was measured using the Zeiss Axiovision 4.0 system and measured at a 45° angle to get the average value.

### Distribution analyses of cortical neuron

The distribution of SATB2^+^ and TBR1^+^ was analyzed according to a previous report (Qian et al., 2020). Briefly, the cerebral organoids were stained for SATB2 and TBR1, and confocal microscopy was used to image random cortical structures in a single z-plane. Radial columns with 100 μm width and 200–500 μm length were taken from the pial surface to cover the complete thickness of the CP region for analysis. The locations of SATB2^+^ and TBR1^+^ were individually labeled on the image, and their y-coordinates were marked and normalized to the CP thickness to analyze their comparative layer location in CP. The frequency distributions of the relative vertical position of each marker in 11 equal bins were determined and plotted in Prism software (GraphPad). To compare the relative number of SATB2^+^ and TBR1^+^ nuclei in each bin, Kolmogorov–Smirnov tests were conducted with a web-based tool.^1^ Cumulative distribution curves were plotted based on the relative positions of all marked nuclei and *p*-values were determined.

### Real-time quantitative PCR

Six to ten organoids were pooled per group and processed as literature’s description (Qian et al., 2020). Briefly, RNA was extracted using kits (QIAGEN). After the quantity and quality were measured, the RNA samples were reverse transcription using the SuperScript III First-Strand Synthesis SuperMix (Invitrogen, United States). RT-qPCR was conducted with Universal SYBR Green Supermix (Bio-Rad, United States) using an CFX Connect^™^ Real-time system (Bio-Rad, United States). The 2exp-ΔΔCt method was used to determine the relative expression of each gene with GAPDH as a reference. The primers for RT-qPCR are listed in the Supplementary Table S2. The amplification conditions were as follows: 95°C, 30 s; following 40 cycles of 95°C, 30 s, 60°C, 30 s.

### Western blotting

For extracting the total protein, 6–10 organoids of each group were collected using RIPA lysis buffer (Beyotime, Shanghai, China), and the concentrations were detected with BCA protein assay (Beyotime, China). An equal amount of each protein sample was separated by 10% SDS-PAGE (80 V, 30 min; 120 V, 80 min) and then transferred onto a PVDF membrane. The membranes were incubated with rabbit anti-β-catenin (1:1000, ab32572, Abcam, United States) and mouse anti-GAPDH (1:1000, ab8245, Abcam, United States) at 4°C overnight followed by anti-rabbit or mouse secondary antibodies for 2 h at room temperature. The band signal was detected using the chemiluminescence detection kit (Bio-Rad, United States) according to the instructions. Band intensities were quantified using Image Lab (Bio-Rad, United States), and the β-catenin protein was normalized to GAPDH.

### Library preparation and sequencing

The human cerebral organoids with different concentrations in the VPA treatment group were collected on days 28, 56, and 84 for the mRNA sequencing assay (six samples for day 28 and four samples for days 56 and 84 in each group). Sequencing libraries were produced, then the library products were purified and the quality was evaluated. Finally, the library samples were clustered and sequenced.

All human cerebral organoids RNA-seq data are available in the NCBI Sequence Read Archive (SRA) through BioProject ID PRJNA802072.

### The correlation analysis of the organoid

The spearman correlation between the three stages of the organoid and the six life stages of the human dorsolateral cortex was calculated (Jaffe et al., 2015). At the same time, the same method was applied to analyze the relationship between organoids and brains with different developmental stages and regions. The human fetal organs were obtained from GSE66302. Fetal tissues aged 8–37 post-conception weeks (PCW) and the brain regions obtained from Allen Brain Atlas (RPKM datasets).

To quantify gene expression correlation between organoids and the human dorsolateral cortex, fetal organs, and fetal tissues. The download datasets filter low-expressed genes to obtain moderately high-expressed genes. Genes with a mean expression level above three and a standard deviation greater than one were obtained from the three downloaded datasets. Spearman correlations were calculated according to the expression levels of these genes. Autism-related risk genes were gained from the online website: Simons Foundation Autism Research Initiative (SFARI).^2^ To pick up autism-related risk genes in our studies, the overlap DEGs from each group was computed using the Venn Diagram package. R programming language was applied to conduct all data analysis and produce the figures.

### Function analysis of the differential expression genes

After filtering the low-quality reads, genes with counts per million value greater than one remained in at least two groups. The data was normalized by the upper quartile algorithm. DEGs between VPA-treated and control groups were identified using the DESeq2 package. Genes with false discovery rate (FDR) < 0.05 and a log (fold-change) > 2were considered DEGs. To investigate the function of DEGs in VPA-treated groups, Gene Ontology (GO) and pathway enrichment analysis of DEGs were implemented by the ToppGene web server. The PPI networks were constructed using the STRING database *via* online NetworkAnalyst web server, and Communities in the PPI network were detected by the visualized using Cytoscape (v3.6) and InfoMap algorithm.

### Statistical analysis

Cerebral organoids were randomly assigned to treatment, all analyses were conducted randomly by experimenters blinded to the treatment group. Statistical analysis was performed using SPSS 20.0 software. Differences between two groups were analyzed by unpaired *t*-test and differences among multi-groups by one-way ANOVA. All results are presented as mean ± SEM and *p* < 0.05 taken as statistically significant. *, **, and *** represent *p*-values <0.05, 0.01, and 0.001 in the graphed data, respectively.

## Results

### VPA exposure inhibited the expansion in human dorsal forebrain organoids

**Figure 1:**
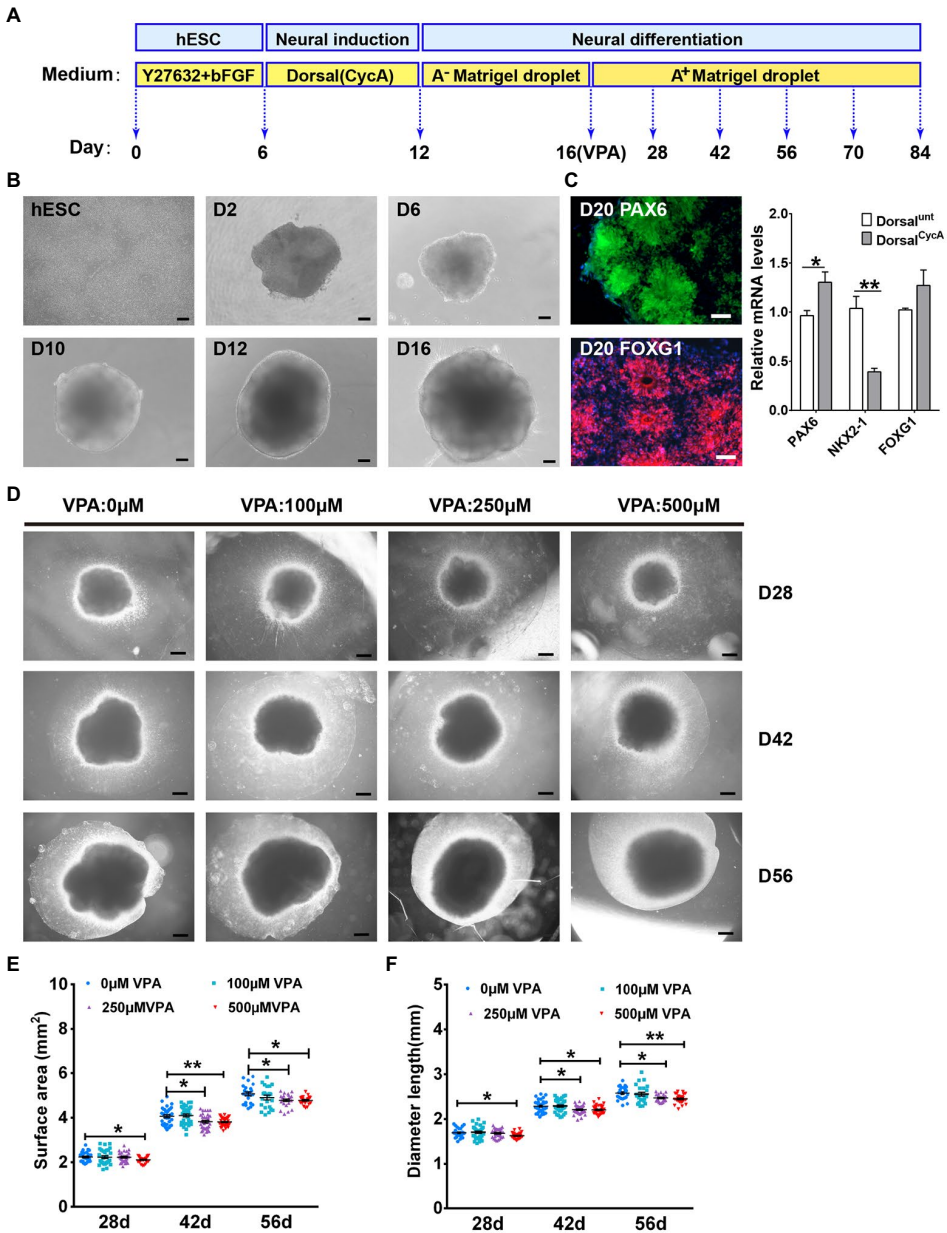
VPA exposure inhibited the expansion in human dorsal forebrain organoids. **(A)** A schematic diagram illustrating the procedures for producing dorsal forebrain cerebral organoids from human hESCs. **(B)** The organoids exhibited large neuroepithelial loops and the overall organoid sizes consistently increased over time. Scale bars, 100 μm. **(C)** The organoids exhibited a dorsal forebrain identity at day 20 (*n* = 3 organoids each). Scale bars, 50 μm. **(D)** Representative pictures from control and VPA treated cerebral organoids at days 28, 42, and 56. Scale bars, 500 μm. **(E,F)** The growth of dorsal cerebral organoid from day 28 to day 56 were further assessed using surface area **(E)** and diameter **(F)** at day 28 (*n* = 29 organoids each), 42 (*n* = 34 organoids each), and 56 (*n* = 23 organoids each). *p  > 0.05, **p > 0.01.

To investigate the effect of VPA exposure on the development of human corticogenesis, we generated human dorsal forebrain organoids following an optimized protocol (Bagley et al., 2017) and VPA was added during the critical period of neuroepithelial expansion (Figure 1A). In the neural induction stage, the organoids were added with CycA, referred to as “dorsal^CycA^,” in contrast to “dorsal^Unt^” organoids generated with an intrinsic patterning protocol (without CycA). We found that organoids exhibited large neuroepithelial loops and the sizes of organoids consistently enlarged over time (Figure 1B). Typically, on day 20, we confirmed the acquisition of a forebrain-specific fate with the expression of forebrain specific FOXG1^+^ (Martynoga et al., 2005) progenitors and FOXG1 mRNA level was similar in both dorsal^CycA^ and dorsal^Unt^ organoid. The dorsal forebrain identity of organoids was shown by immunostaining for marker PAX6 (Georgala et al., 2011). Meanwhile, PAX6 mRNA level was increased in dorsal^CycA^ organoids compared to dorsal^Unt^ organoids, while ventral forebrain marker NKX2-1 (Sussel et al., 1999) was decreased (Figure 1C). Immunohistological staining of dorsal organoid sections showed NPCs markers NESTIN, PAX6, Vimentin, and SOX2 expressing in the rosette, MAP2-labeled neurons well positioned near the ventricle-like structures (Supplementary Figures S1A–C). By day 28, we observed a well-defined polarized neuroepithelium-like structure labeled SOX2^+^ and PKCλ^+^ as well as a consistent ratio between SOX2^+^/BLBP^+^ progenitor layer in dorsal^CycA^ organoids compared to dorsal^Unt^ organoids (Supplementary Figures S1D–F). The cerebral organoids showed proliferation markers P-H3, Ki67, and p-Vimentin, and intermediate progenitor (IP) cell marker TBR2 (Supplementary Figures S1G–L) at days 28 and 42, stratified neuroepithelium-like cortical layer marker TBR1 at day 56 (Supplementary Figure S1M). We found that stratified GFAP-positive astrocytes emerged in the cortical plate-like (CP) layer at days 56 and 70 (Supplementary Figures S1N,O), and typical mature astrocytes labeled with S100β scattered at day 84 (Supplementary Figure S1P).

The apparent homogeneity of dorsal forebrain organoids with well-developed cortical structure is amenable to testing the effects of VPA. At day 28, surface area and diameter were indistinguishable in the organoids with VPA treatment at 100 and 250 μM compared with control organoids. However, the organoids with 500 μM VPA treatment displayed markedly smaller surface area and diameter than control organoids (Figures 1D–F). At days 42 and 56, both 250 and 500 μM VPA treated organoids were markedly decreased in the surface area and diameter compared with control organoids, whereas alterations were not detected in the organoids with 100 μM VPA treatment (Figures 1D–F). These results suggest that VPA treatment induced a reduction in the organoid size in dose-dependent manner.

### Cerebral organoids faithfully recapitulate human cortical development

**Figure 2:**
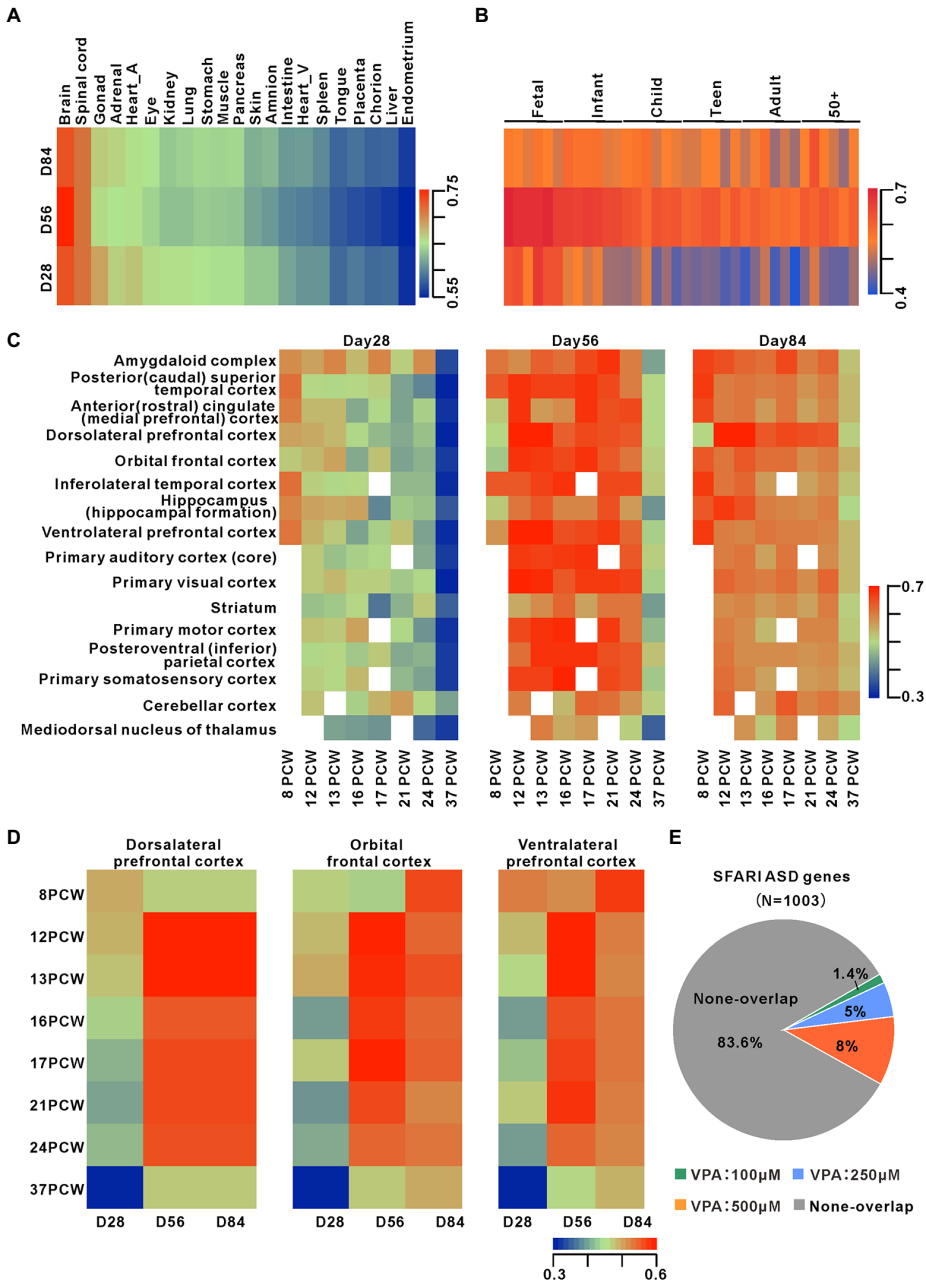
Transcriptomes comparison between dorsal forebrain organoids and human fetal brain Development. **(A)** Heatmap of Pearson’s correlation analysis of RNA-seq datasets from days 28 (*n* = 6 organoids each), 56 (*n* = 4 organoids each), and 84 (*n* = 4 organoids each) organoids and datasets for 21 human fetal organs. The figure presented the mean values of biological replicates. **(B)** Pearson correlation heatmap among organoids at early stages and human dorsolateral prefrontal cortex samples from 6 life stages (Jaffe et al., 2015). **(C)** Pearson correlation heatmap among organoids and transcriptome datasets from 16 different cortical regions across 8 early human stages (http://www.brain-map.org/). **(D)** Pearsoncorrelationheatmap among organoids and transcriptome datasets from 3 different cortical subregions across 8 early human stages. **(E)** Overlap of DEGs during organoid development with ASD-related risk genes (from https://gene.sfari.org/autdb/HG_Home.do). The statistically significant overlapping genes were determined (*p* < 0.001, chi-square test).

To assess the dorsal forebrain specificity as well as the maturity of organoids, the global transcriptomes from days 28, 56, and 84 organoids were analyzed by RNA-seq. We compared these organoid transcriptional profiles with data sets from 21 different human fetal organs (Roost et al., 2015). Pearson’s correlation analysis suggested significant correlations between the organoids from three-time points and fetal brains and spinal cord (Figure 2A). Comparison to the transcriptomes from human dorsolateral prefrontal cortex samples across six life stages, revealed these organoids are the highest correlation with fetal brain tissues, day 56 organoids with the best correlation (Figure 2B). Therefore, these data indicate that the development of the cultured organoids is linked to developing fetal brain at the molecular level.

For determining the stage of forebrain organoid development and the characteristics of brain sub-regions, we compared transcriptome datasets of 16 brain regions of human brain regions at eight life stages (Figure 2C). Data showed that day 28 organoid profiles were highly associated with several cortical regions at eight post-conception weeks (PCW) and profiles at days 56 and 84 organoids were more highly associated with 12–24 PCW in multiple cortical areas. The comparisons of organoid profiles with transcriptomes of the human dorsolateral prefrontal cortex, orbital frontal cortex, and ventrolateral prefrontal cortex showed a temporal correlation between our preparation and the development of the dorsolateral prefrontal cortex (Figure 2D). To further confirm the successful dorsal lateralization of cerebral organoid tissue，we examined the expression of specific markers of day 28 organoids. We found that higher levels of the forebrain marker FOXG1 and the dorsal forebrain markers PAX6 and TBR1 (Hevner et al., 2011) in day 28 organoids, while the lower levels of ventral forebrain markers NKX2-1, DLX2 (Hernández-Miranda et al., 2010), GSX2(Kessaris et al., 2014), weak levels of mid-hindbrain makers ATOH1 and WNT1, and HOX genes such as HOXB2, HOXB3, or HOXA2 (Rosebrock et al., 2022; Supplementary Figure S2). There is a link between gestational exposure to VPA and the risk of autism in their offspring, so we compared organoid transcriptional profiles with three gene sets related to ASD risk. These results showed 14, 50, and 80 genes overlapping with 100, 250, and 500 μM from the SFARI database (Figure 2E; Supplementary Figure S3). Moreover, these three different concentrations of VPA-caused DEGs markedly overlapped with irregulated genes in PsychENCODE ASD brains (Overlapped genes = 16, 65, 123) and organoids from ASD subjects (Mariani et al., 2015; Overlapped genes = 83, 219, 271; Supplementary Figures S3). Notably, 4 DEGs such as GABRB2, SCN1A, GRID2, and SLC24A2, shared in the three ASD gene sets with 250 and 500 μM VPA. Thus, the dorsal forebrain organoids can be a good model for exploring the roles of dynamic development expression of ASD risk genes in the human brain.

These results suggest that the human dorsal forebrain organoids are similar to the cortical development of human embryos *in vivo* and thus indicate that these organoids can be a good model for understanding the effects of early treatment of VPA on human cortical development.

### VPA exposure decreased proliferation and expansion of NPCs in human dorsal forebrain organoids

**Figure 3:**
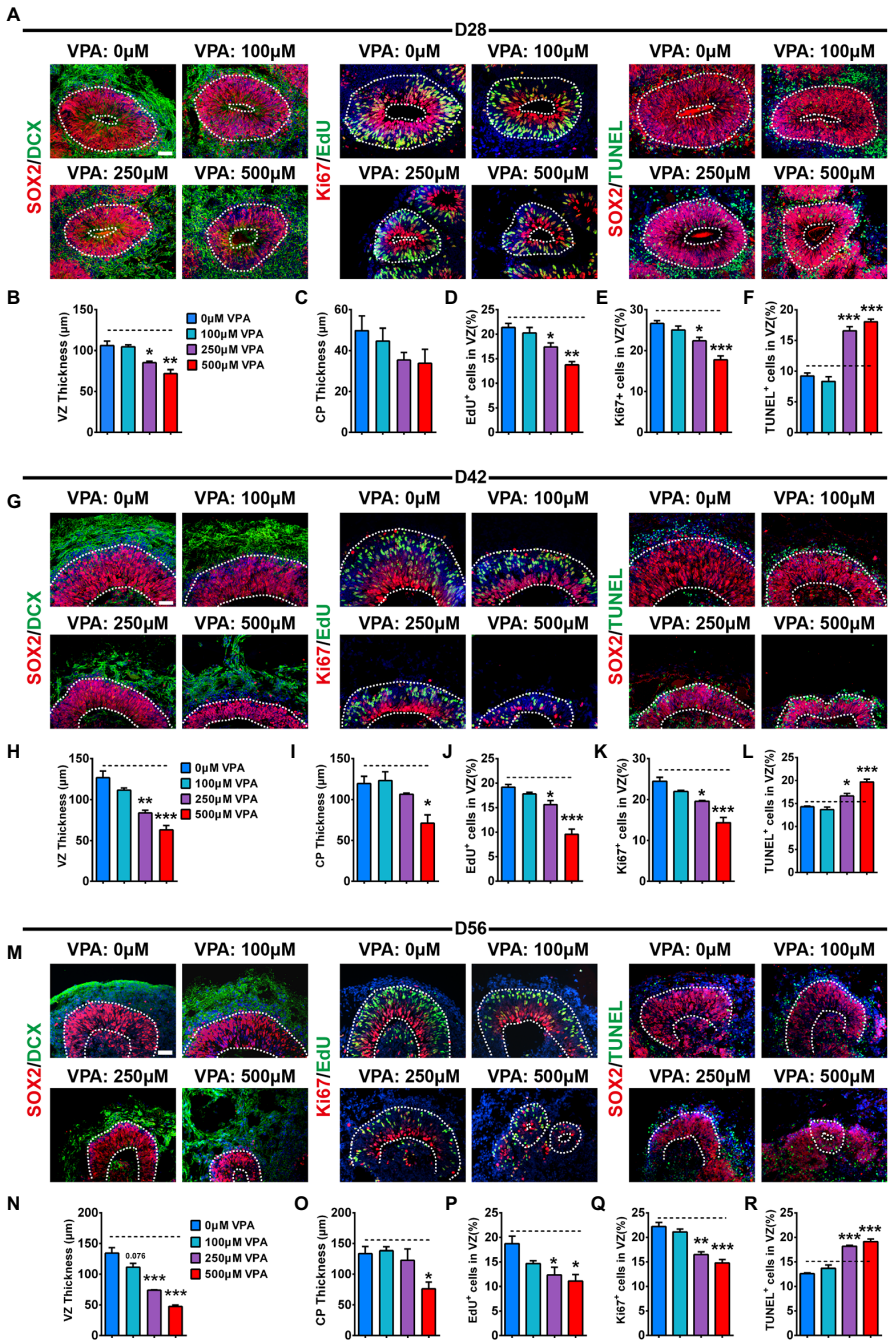
VPA exposure decreased proliferation and expansion of NPCs in human dorsal forebrain organoids. Immunostaining for SOX2 (red) and DCX (green), EdU (green) and ki67 (red), SOX2 (red) and TUNEL (green) of days 28 **(A)**, 42 **(G)**, and 56 **(M)** organoids (*n* = 3 organoids each). Quantification of the SOX2^+^ VZ thickness **(B,H,N)** and DCX^+^ CP thickness **(C,I,O)** of organoids at days 28 **(B,C)**, 42 **(H,I)**, and 56 **(N,O)**. Quantification of the percentage of EdU^+^ **(D,J,P)** and Ki67^+^ **(E,K,Q)** cells in VZ-like regions of organoids at days 28 **(D,E)**, 42 **(J,K)**, and 56 **(P,Q)**. **(H)** Quantification of the ratio of TUNEL^+^ cells in VZ-like regions of organoids at days 28 **(F)**, 42 **(L)**, and 56 **(R)**. White dotted lines mark SOX2^+^ VZ, and DCX^+^ CP-like areas. Scale bars, 50 μm. *p  > 0.05, **p  > 0.01, ***p  > 0.001.

To explore the pathological mechanism of VPA causing a reduction in the sizes of dorsal^CycA^ organoids, we further analyzed the alterations in the NPCs pool. SOX2, a marker for apical NPCs, is also used to quantify the thickness of the ventricular zone (VZ). We noticed well-organized VZ-like structures with dense SOX2 stained NPCs near the lumen at days 28, 42, and 56 organoids (Figures 3A,G,M). Consistent with reduced organoid size, 250 and 500 μM VPA treatment caused a substantial reduction in the VZ thickness at days 28, 42, and 56 organoids, whereas 100 μM VPA treated organoids were not significantly altered in the VZ thickness. A slight decrease trend was observed at day 56 organoids (Figures 3B,H,N). It suggests a reduction of the NPCs pool in the higher-dose VPA-treated organoids. A previous study showed that robust NPCs differentiation quantified with the relative thickness of the CP layer was observed at day 28 (Qian et al., 2016). VPA treatment did not delay neuronal differentiation at day 28, reflected by the comparable expression of DCX in these organoids (Figure 3C). This has been further confirmed by the quantification of immature neuronal proteins TUJ1, MAP2, and DCX (Supplementary Figure S4). We noticed that 100 and 250 μM VPA treatment did not alter CP thickness at days 42 and 56, whereas 500 μM VPA treatment caused a significant reduction (Figures 3I,O). It is supposed that 500 μM VPA treatment delayed the neural differentiation related to a decrease in the NPCs pool. The proliferation of NPCs was detected with EdU and Ki67. EdU pulse experiments showed a marked decrease in the ratio of EdU^+^ cells in the VZ-like regions of 250 and 500 μM VPA treated organoids at days 28, 42, and 56 (Figures 3D,J,P). Immunostaining of Ki67 revealed that 250 and 500 μM VPA treated organoids displayed a decreased percentage of cycling cells in the VZ (Figures 3E,K,Q). However, 100 μM VPA treatment did not affect the ratios of EdU^+^ (Figures 3D,J,P) and Ki67^+^ (Figures 3E,K,Q) cells in the VZ significantly. We further observed significantly increased TUNEL^+^ cells in the VZ-like regions of 250 and 500 μM VPA treated organoids at days 28, 42, and 56 (Figures 3F,L,R). The organoids treated with 100 μM VPA showed no statistical difference in the ratios of TUNEL^+^ cells in the VZ at days 28, 42, and 56 compared to control organoids (Figures 3F,L,R). Therefore, these results suggested that a higher dosage of VPA treatment decreased the NPCs pool due to the inhibition of the proliferation of NPCs and promotion of its apoptosis.

### Enrichment of Wnt signaling pathway activation involved in the reduction of NPCs pool in the VPA treated organoids

**Figure 4:**
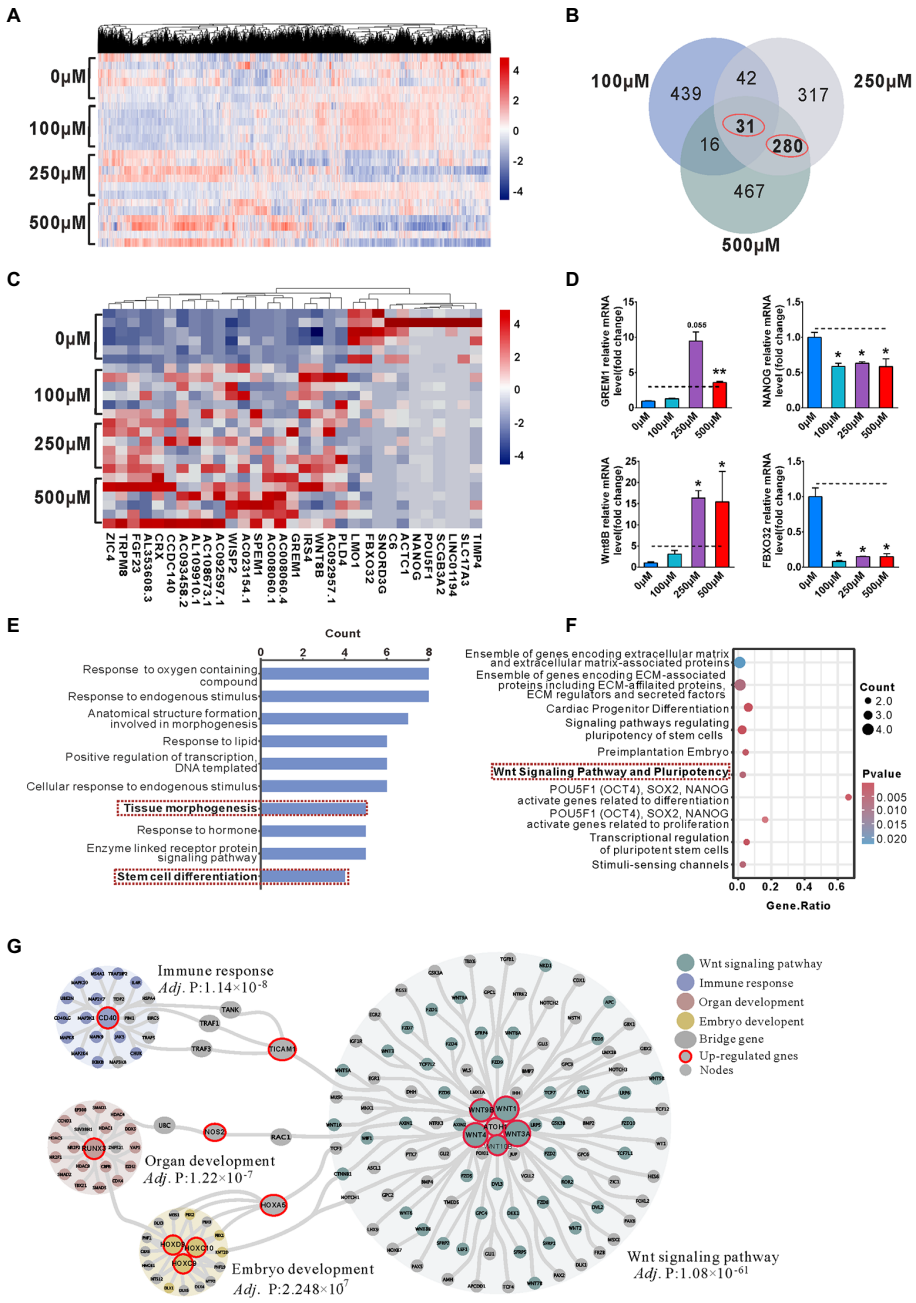
Enrichment of Wnt signaling pathway activation involved in the reduction of NPCs pool in the VPA treated Organoids. **(A)** Hierarchical clustering heatmap for DEGs from the organoids of control (0 μM) and VPA treated organoids (100, 250, and 500 μM) on day 28 (*n* = 6 organoids each). **(B)** Venn diagram of co-regulated DEGs by VPA. **(C)** Hierarchical clustering heatmap for the co-regulated 31 DEGs. **(D)** The qRT-PCR was conducted to confirm the results of RNA-seq (*n* = 3 organoids each). **(E)** Enriched top 10 GO-biological process terms. **(F)** Top 10 enriched pathways. **(G)** PPI network analyses screened out four main communities. *p  > 0.05, **p > 0.01.

To further address the transcriptome profiles related to the phenotype, we performed bulk RNA-seq on 4-week-old organoids. Hierarchical clustering heatmap showed the gene expression profile in 0, 100, 250, and 500 μM VPA treated organoids (Figure 4A). We identified 528, 670, and 794 differentially expressed genes (DEGs) between the 100 and 0 μM, 250 and 0 μM, and 500 and 0 μM, respectively, with an overlap of 31 genes (Figure 4B). Hierarchical clustering analysis of the 31 co-regulated genes showed the VPA treatment caused similar effects on early organoid development (Figure 4C). The relative levels of selected most significantly changed genes (*FBXO32*, *NANOG*, *Wnt8B*, and *GREM1*) were further verified by RT-qPCR (Figure 4D). Next, Gene Ontology (GO; Figure 4E) and pathway (Figure 4F) enrichment were conducted on 31 DEGs. GO enrichment showed marked enrichment for genes involved in several biological processes such as anatomical structure formation involved in morphogenesis, tissue morphogenesis and stem cell differentiation (Figure 4E). In accordance, significant enrichment in pathway analysis showed to be associated with Wnt signaling pathway and pluripotency, signaling pathways regulating pluripotency of stem cells, and transcriptional regulation of pluripotent stem cells (Figure 4F).

Hierarchical clustering analysis of 280 genes co-regulated by 250 and 500 μM VPA treatment shows that the higher doses of VPA treatment had similar effects on early organoid development (Supplementary Figure S5A). We found that 480 and 560 genes were up-regulated, 190 and 234 down-regulated by 250 and 500 μM VPA treatment (Supplementary Figure S5B). GO enrichment revealed significant enrichment for genes involved in the “regulation of cell population proliferation,” suggesting a potential involvement of higher doses of VPA in the inhibition of NPCs proliferation (Supplementary Figures S5C,D). PPI network of the co-regulated 280 DEGs was shown (Figure 4G). The genes were classified into three groups by community detection: hub genes, bridge genes, and node genes. Consequently, four main communities were defined with nodes ≥18 and an *adj*. *p* value <0.0001 (extremely significant level). In the network, genes with a degree ≥8 were considered as hub genes in each community. Based on the degree of each node in each community, there were 11 up-regulated hub genes, *Wnt3A* (degree = 77), *Wnt1* (degree = 71), *Wnt4* (degree = 69), *Wnt9B* (degree = 50), *CD40* (degree = 31), *RUNX3* (degree = 24), *Wnt10B* (degree = 20), *ATOH1* (degree = 14), *HOXD9* (degree = 10), *HOXC9* (degree = 8), and *HOXC10* (degree = 8). The three up-regulated hub genes (*TICAM1*, *NOS2*, and *HOXA5*) were connected to different communities. Hierarchical clustering heatmap showed the hub gene mRNA expression profile in 0, 100, 250, and 500 μM VPA treated organoids (Supplementary Figure S6). Interestingly, four communities such as the Wnt signaling pathway, immune response, organ development, and embryo development were affected by up-regulated hub genes. In particular, the Wnt signaling pathway community contains the most pathway-associated genes, supposed to contribute to maintaining the NPCs pool within the early cerebral organoid development.

### VPA exposure decreased abundance of progenitor cells and neurons in cerebral organoids at 56

**Figure 5:**
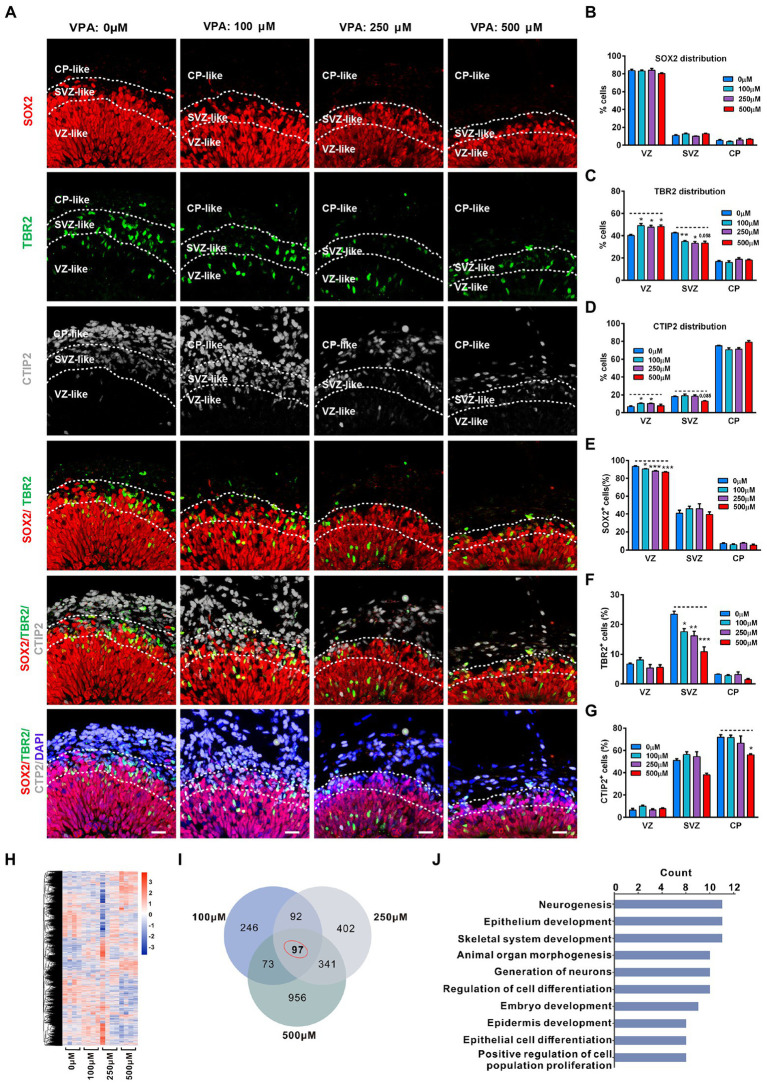
VPA exposure decreased abundance of progenitor cells and neurons in human dorsal forebrain organoids at day 56. **(A)** Representative image of 0, 100, 250, and 500 μM VPA treated human dorsal forebrain organoids at day 56 (0 μM, 100 μM: *n* = 4 organoids; 250 μM: *n* = 5 organoids; 500 μM: *n* = 3 organoids). The defined VZ, SVZ, and CP-like regions are delineated according to the combination of DAPI, SOX2, TBR2, and CTIP2 staining patterns. Scale bars, 20 μm. **(B–D)** Analysis of marker allocation across the VZ/SVZ/CP within the organized regions in 0, 100, 250, and 500 μM VPA treated human dorsal forebrain organoids. **(E–G)** Analysis of the percentage of SOX2^+^, TBR2^+^, and CTIP2^+^ cells out of total cells in the examined areas. Scale bars, 20 μm. **(H)** Hierarchical clustering heatmap for DEGs from the organoids of control (0 μM) and VPA treated organoids (100, 250, and 500 μM) on day 56 (*n* = 4 organoids each). **(I)** Venn diagram of co-regulated DEGs by VPA. **(J)** Enriched top 10 GO pathways in biological process. *p  > 0.05, **p  > 0.01, ***p  > 0.001.

To further observe the consequence of the reduction of the NPCs pool, the distributions of NPCs and neurons in 56-day organoids were analyzed. At this stage, staining of RG marker SOX2, IP cell marker TBR2, and deep cortical layer marker CTIP2 showed three separate regions, similar to developing VZ, SVZ, and CP. These regions can be distinguished by cell number, cell direction, and expression levels of SOX2, TBR2, and CTIP2. Therefore, VZ was determined by the packed SOX2 stained RG cells, while SVZ was recognized based on scattered and horizontal cells expressing TBR2. Close to the border of TBR2 is the original CP, and CTIP2 is strongly expressed (Figure 5A). Comparable distribution of SOX2 was detected in organoids from four groups, with the majority (≈80%) of SOX2^+^ cells in the VZ-like regions (Figure 5B). VPA treatment induced a notable increase in IP cell distribution in the VZ-like region whereas a reduction in the SVZ-like region (Figure 5C). Both 100 and 250 μM VPA treatment increased the distribution of CTIP2 in the VZ-like region, but 500 μM VPA treatment caused a decreased trend of CTIP2 distribution in the SVZ-like region (Figure 5D). These results indicated that the layer distribution pattern of the cell types was affected by VPA treatment in a dose-dependent fashion. In addition, analysis of the relative cell proportion revealed that VPA treatment caused a significant decrease in the SOX2^+^ cells in the VZ-like regions and IP cells in the SVZ-like regions (Figures 5E,F). We also noticed that 500 μM VPA treatment reduced the CTIP2^+^ neurons in the CP-like regions (Figure 5G). These results suggested that VPA exposure dampened the migration of IP cells and early born neurons.

To further examine the underlying mechanism of the effect of VPA treatment, we conducted RNA-seq in organoids of 0, 100, 250, and 500 μM VPA treated organoids at 56 days. The hierarchical clustering heatmap showed gene expression profiles in the whole organoid tissue of four groups (Figure 5H). Venn diagram showed there were 508, 932, and 1,467 DEGs for 100, 250, and 500 μM VPA treated organoids compared with controls, respectively, and 97 genes that were co-regulated by VPA treatment among these DEGs (Figure 5I). Next, GO enrichment was performed on these 97 DEGs and the top 10 biological processes related to neurogenesis, generation of neurons, regulation of cell differentiation, embryo development, and positive regulation of cell population proliferation (Figure 5J). Consistent with the distribution of progenitor cells and neurons, this might infer that reduction in the pool of NPCs caused an ultimately decreased output of IP cells, and 500 μM VPA treatment also inhibited neuron production in the cerebral organoids.

### VPA exposure caused disorganization of cortical lamination and loss of upper-layer neurons in dorsal forebrain organoids

**Figure 6:**
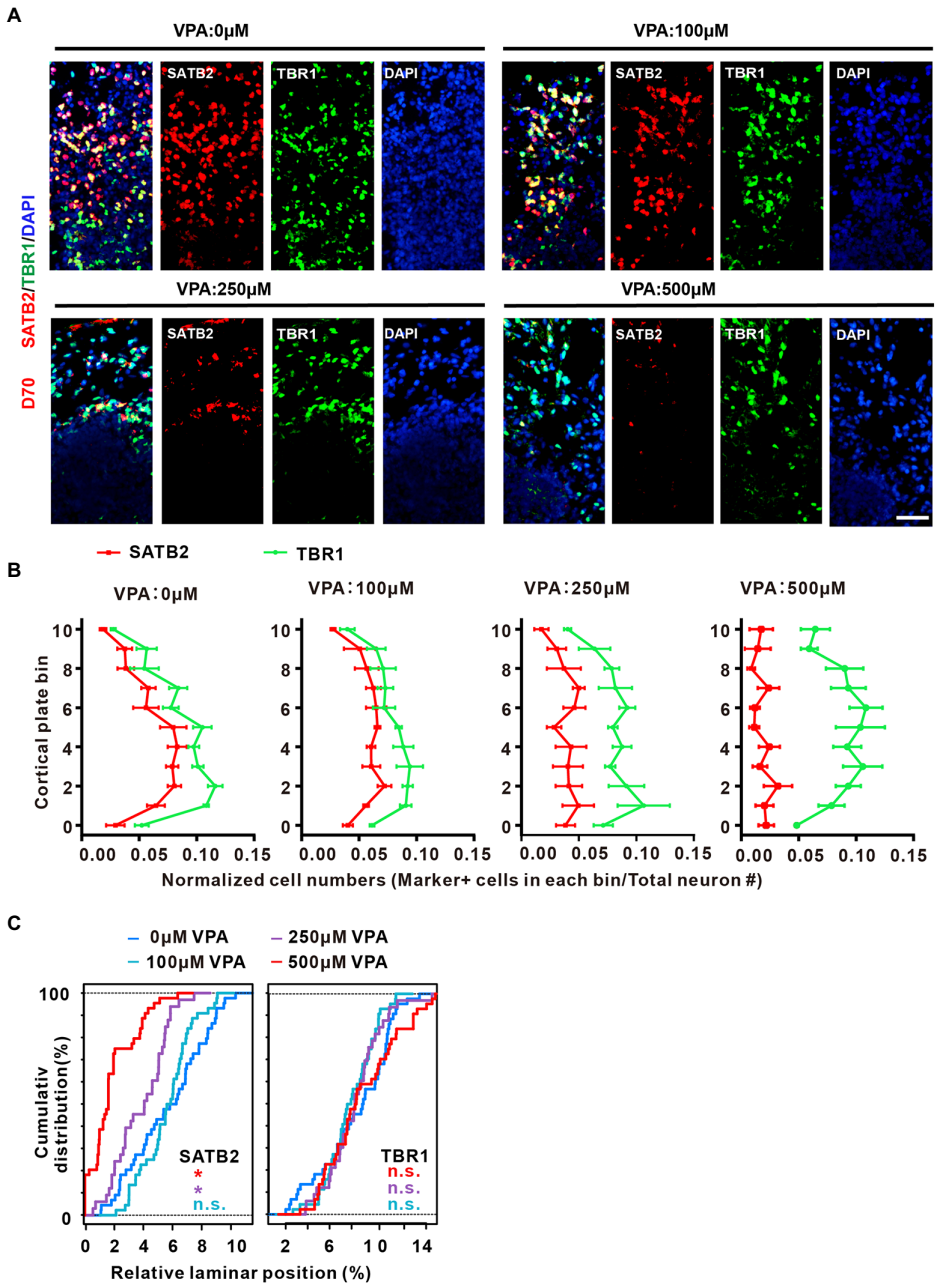
VPA exposure caused disorganization of cortical lamination and loss of up-layer neurons in dorsal forebrain organoids. **(A)** Representative image of 0, 100, 250, and 500 μM VPA treated human dorsal forebrain organoids for SATB2 and TBR1 at day 70 (0, 100, and 500 μM: *n* = 4 organoids; 250 μM, *n* = 3 organoids). Scale bar, 50 μm. **(B)** Quantifications of the location of SATB2^+^ and TBR1^+^ cells in the CP of human dorsal forebrain organoids with different concentrations of VPA. The CP area is equally divided into 11 equal bins; bins 0–10 follow the direction from apical to basal. The curves indicate the normalized abundance in each bin, computed as marker^+^ cell number in a bin/total neuron number. **(C)** Kolmogorov–Smirnov tests were conducted for the VPA-exposed conditions, referred as the corresponding color, against the 0 μM condition (n.s.: *p* > 0.05; *p < 0.05).

To further explore the decreased IP cells and neurons as a consequence of the organization of cortical lamination, we examined the distribution of the deep-layer neuron marker TBR1 and the upper-layer neuron marker SATB2 in the CP-like regions of day 70 organoids. Similar to a previous report (Qian et al., 2020), the locations of SATB2 and TBR1 in the CP were intermingled without layer preferences (Figure 6A). The thickness of the CP in 11 evenly spaced bins was used to analyze the laminar expression patterns. VPA-treated organoids at different concentrations showed comparable TBR1 expression in the cortical bins compared with control organoids. In contrast, 250 and 500 μM VPA treated organoids showed decreased SATB2 expression across the entire CP, although 100 μM VPA treatment did not alter the SATB2 expression in cortical bins (Figures 6B,C). These results revealed that 250 and 500 μM VPA treatment impaired neuronal fate specification of upper layers in the CP of cultured organoids.

### VPA exposure decreased oRG-like cell production in human dorsal forebrain organoids

**Figure 7:**
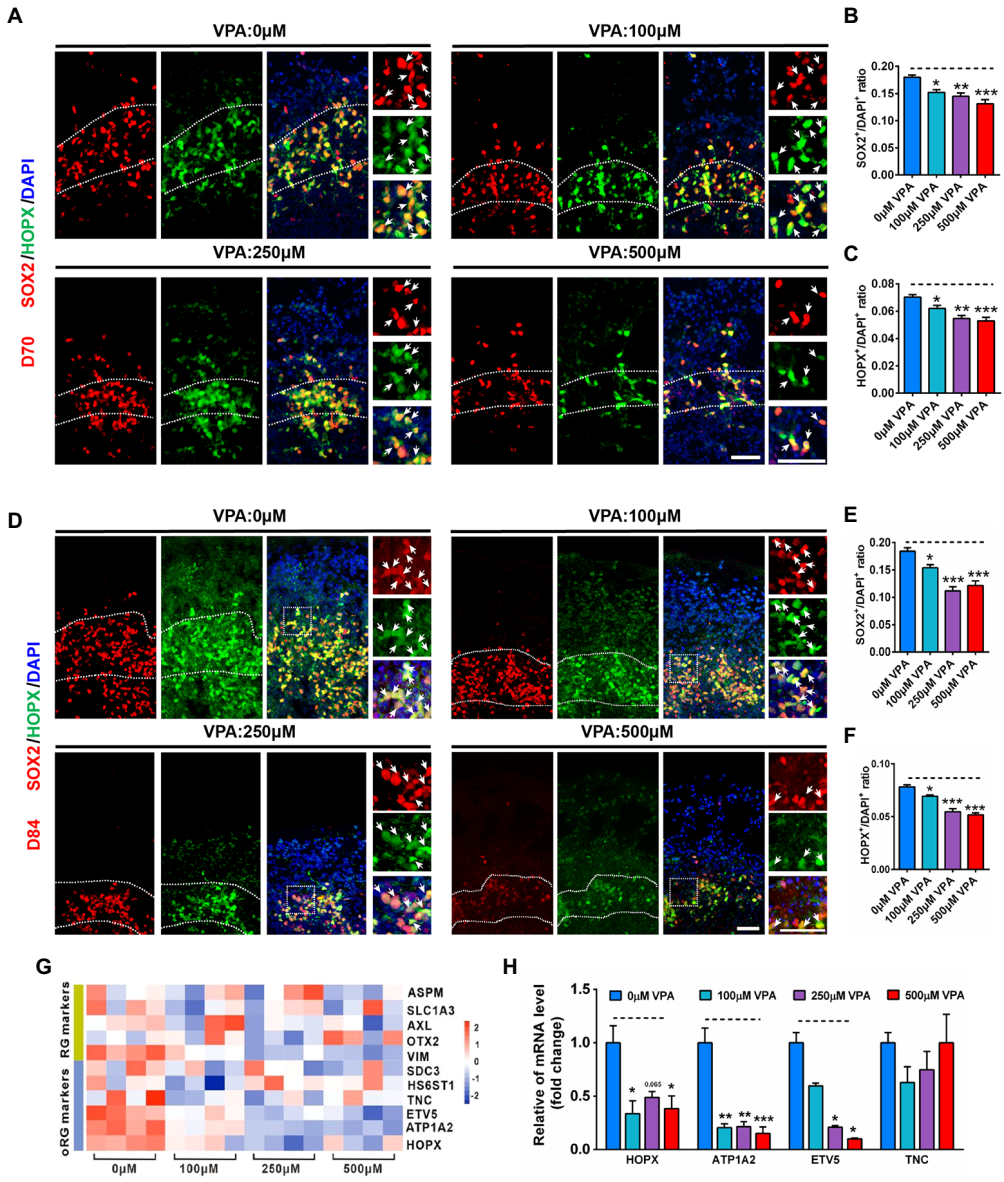
VPA exposure decreased oRG-like cell production in human dorsal forebrain organoids. **(A)** Representative image of 0, 100, 250, and 500 μM VPA treated human dorsal forebrain organoids for SOX2 (red) and HOPX (green) at day 70. Scale bars, 50 μm. **(B,C)** Quantification of the ratios of SOX2^+^/DAPI^+^ and HOPX^+^/DAPI^+^ in the oSVZ-like regions of organoids at day 70 (*n* = 4 organoids each). **(D)** Representative images of 0, 100, 250, and 500 μM VPA treated human dorsal forebrain organoids for SOX2 and HOPX at day 84. Scale bar, 50 μm. **(E,F)** Quantification of the ratios of SOX2^+^/DAPI^+^ **(E)** and HOPX^+^/DAPI^+^ **(F)** in the oSVZ-like regions of organoids at day 84 (*n* = 4 organoids each). **(G)** Heatmap displaying relative gene expression of marker genes (RG identity markers, oRG markers) of 0, 100, 250, and 500 μM VPA treated human dorsal forebrain organoids (*n* = 4 organoids each). **(H)** RT-qPCR were conducted to confirm the results of RNA-seq of **(G)** (*n* = 3 organoids each). *p  > 0.05, **p  > 0.01, ***p  > 0.001.

Typically, oSVZ-like progenitor layers formed by oRGs are the dominant NPCs generating upper-layer cortical neurons in the developing human cortex (Smart et al., 2002; Lukaszewicz et al., 2005). The radial scaffolds are essential for maintaining the cytoarchitecture of both NPCs layers and CP and guide newly generated neurons migrating along with the radial processes of oRGs (Uzquiano et al., 2018). It has been shown that the oRG cells emerge at day 70, and are increased with the development over time (Bershteyn et al., 2017). Indeed, we can detect SOX2^+^/HOPX^+^ double-stained oRG cells in the VZ as early as day 56 (Supplementary Figure S7). At day 70, the organoids contained lots of oRG cells located in the oSVZ-like layer (Figure 7A), and VPA treatment reduced the ratios of SOX2^+^/DAPI^+^ and HOPX^+^/DAPI^+^ (Figures 7B,C). At day 84, the organoids contained a distinct SOX2^+^/HOPX^+^ oSVZ-like layer exhibiting unipolar morphology of oRGs with radially oriented basal processes toward the pial surface (Figure 7D). VPA treatment caused a sharp reduction in the thickness of the oSVZ-like structure. A sharp shrinkage in the thickness of the oSVZ-like structure labeled by SOX2^+^/HOPX^+^ was reduced by VPA treatment. The ratios of SOX2^+^/DAPI^+^ and HOPX^+^/DAPI^+^ were decreased by VPA treatment, suggesting VPA treatment influenced the generation of oRGs and oSVZ-like structures (Figures 7E,F).

To further examine the underlying mechanism of the effect of VPA on oRG, we performed RNA-seq in whole organoids of 0, 100, 250, and 500 μM VPA treatment. Several genes associated with RG and oRG markers were down-regulated in VPA-treated organoids, especially the oRG markers (Figure 7G). To validate the RNA-Seq data, four selected oRG markers such as *HOPX*, *ATP1A2*, *ETV5*, and *TNC* were further confirmed by RT-qPCR (Figure 7H). Venn diagram showed 54 genes were co-regulated by VPA among these DEGs (Supplementary Figure S8B). Hierarchical clustering analysis of the 54 co-regulated genes revealed VPA treatment had similar effects on the co-regulated genes (Supplementary Figures S8A). GO enrichment on these 54 DEGs showed several biological processes associated with reproduction and embryo development that were significantly altered (Supplementary Figure S8D). Accordingly, significant enrichment in pathway analysis was found to be related to the NPCs proliferation (SOX2, OCT4, and NANOG) pathway (Supplementary Figure S8C). Collectively, our findings implicate higher dose of VPA delays oRG cell generation and highlight abnormal alteration of oRG cell involvement in the development of human ASD.

### Wnt/β-catenin inhibition rescued disruption of neurogenesis in VPA treated organoids

**Figure 8:**
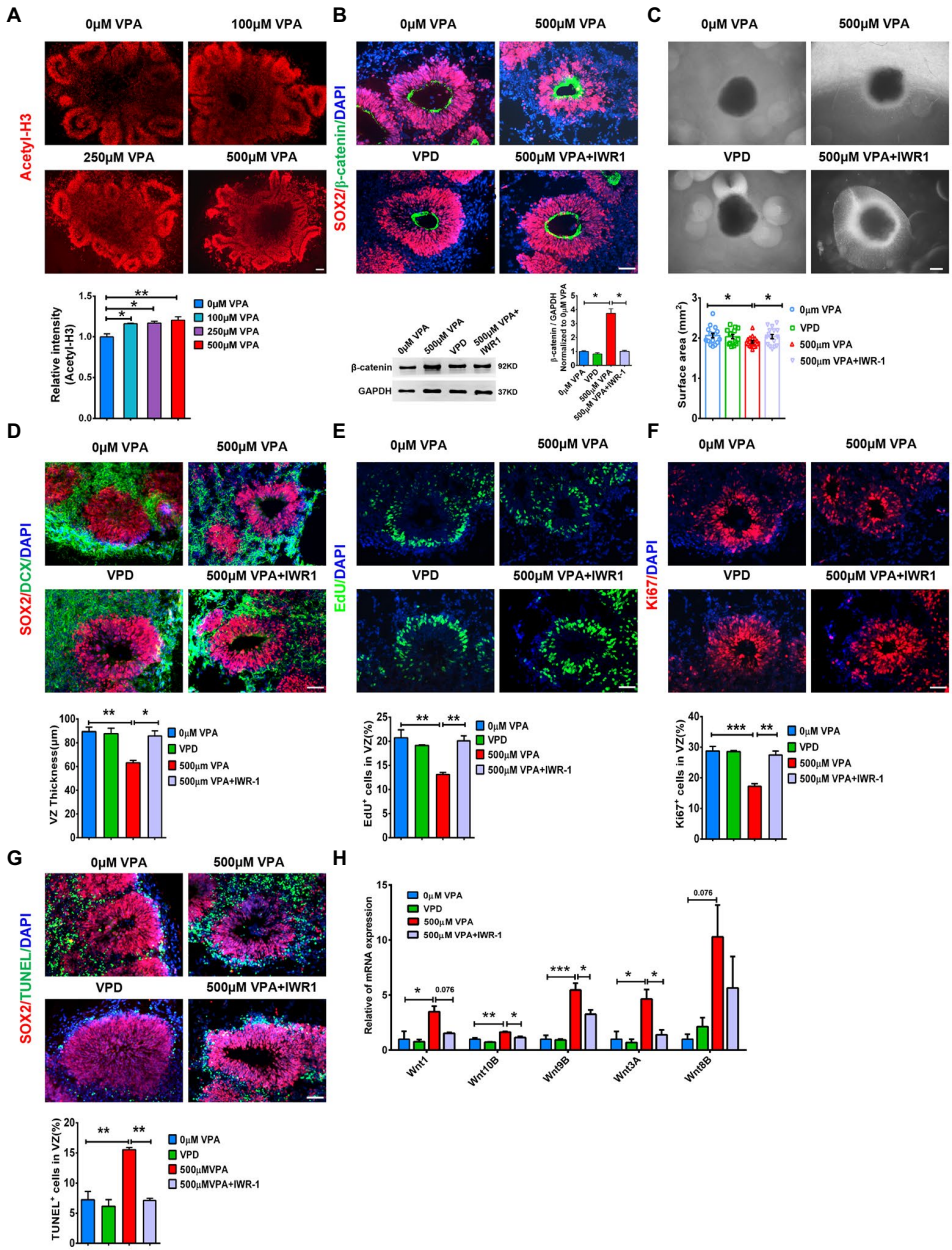
Wnt/β-catenin inhibition rescued deficits in neurogenesis in VPA treated organoids. **(A)** Representative image of 0, 100, 250, and 500 μM VPA treated human dorsal forebrain organoids and quantification of acetyl-H3 staining at day 28 (*n* = 3 organoids each). Scale bar, 100 μm. **(B)** Representative images of SOX2 (red) and β-catenin (green) staining in the cerebral organoids and quantification of β-catenin protein using western blotting at day 28 (*n* = 3 organoids each). Scale bar, 50 μm. **(C)** Representative images of organoids with 0 μM VPA, 500 μM VPA, 1 mM VPD, and 500 μM VPA + 10 μM IWR-1 treatment and quantification of surface areas of the cerebral organoids at day 28 (0 μM, 500 μM VPA, 500 μM VPA + 10 μM IWR-1: *n* = 16 organoids, 1 mM VPD: *n* = 14 organoids). Scale bar, 500 μm. **(D)** Representative images of SOX2 (red) and DCX (green) and quantification of VZ thickness (*n* = 3 organoids each). Scale bar, 50 μm. **(E,F)** Immunostaining for EdU and ki67 and quantification of the percentage of EdU^+^ **(E)** and Ki67^+^ cells **(F)** in VZ-like regions (*n* = 3 organoids each). Scale bar, 50 μm. **(G)** Immunostaining for SOX2 (red) and TUNEL (green) and quantification of the percentage of TUNEL^+^ cells in VZ-like regions (*n* = 3 organoids each). Scale bar, 50 μm. **(H)** The expression levels of five *Wnt* genes were quantified by RT-qPCR (*n* = 3 organoids each). *p  > 0.05, **p  > 0.01, ***p  > 0.001.

VPA, a histone deacetylase (HDAC) inhibitor, generates pathogenic effects during developmental stages (Göttlicher et al., 2001). Consistent with the inhibition of HDAC of VPA action, VPA treatment elevated the level of Acetyl-H3 in cerebral organoids significantly compared to controls (Figure 8A). RNA-seq data showed that early VPA exposure impaired the NPCs pool by activating the Wnt pathway (Supplementary Figure S9). To evaluate whether activation of the Wnt pathway and inhibition of HDAC might contribute to VPA-induced phenotype, we treated day 16 organoids with 0 μM VPA, 500 μM VPA, 1 mM valpromide (VPD, a derivative of VPA, but no acetylation site), 500 μM VPA combined with β-catenin inhibitor IWR-1, and detected whether these drugs reproduce or rescue VPA-induced morphological deficits. We found that 500 μM VPA sharply increased the β-catenin level, which can be inhibited by IWR-1 (Figure 8B). We further confirmed that VPA-mediated inhibition of early neurogenesis could not be replicated by the treatment of VPD. Moreover, blockers of the Wnt pathway efficiently prevent VPA-induced reduction in the surface area and VZ-like thickness (Figures 8C,D) and deficits in NPCs proliferation (Figures 8E,F). We also examined the apoptotic cells with TUNEL in the VZ-like regions. The results showed that IWR-1 also rescued the apoptosis of NPCs, while exposure to VPD did not affect the VZ-like thickness (Figure 8G). RT-qPCR verified that IWR-1 inhibited the increased mRNA levels of Wnt1, Wnt10B, Wnt9B, and Wnt3A in the cerebral organoid caused by 500 μM VPA treatment (Figure 8H). These data indicate that activation of the Wnt pathway and a decrease in HDAC activity maybe play roles in VPA depleting the pool of NPCs.

## Discussion

Here, we used dorsal forebrain organoids produced from hESCs to explore the toxic effects of VPA exposure on cortical early development. We found that VPA inhibited proliferation and promoted cell death of SOX2-positive NPCs and other dividing cells, causing a typical reduction in the production of oRG and upper-layer neurons. This reduction in the cortical cell number contributes to a decrease in gross volume in higher doses of VPA-treated organoids. Bulk RNA-seq analysis displayed the aberrant expression of ASD risk genes and the enrichment of these DEGs for NPCs proliferation and differentiation in cerebral organoids after exposure to VPA. Additionally, the phenotypes of VPA-treated cerebral organoids were dose-dependent, consistent with a higher risk of dose-dependent VPA induced congenital malformations in human newborns (Chanda et al., 2019). We further showed that VPA caused dose-dependent inhibition of cortical neurogenesis due to abnormal Wnt signaling activation, which is responsible for oRG output. Our studies point to new insights into the pathogenesis of VPA exposure-induced ASD.

Several studies reported that VPA treatment caused the inhibition of NPCs proliferation and promotion of neuronal differentiation in primary cell cultures from the hippocampus and cerebral cortex (Hsieh et al., 2004; Fujiki et al., 2013). There is also a contradictory influence of prenatal VPA on NPCs in the rodent brain. Rat embryos exposed to VPA at E13.5 displayed dampened proliferation of NPCs in the intermediate zone of the E15.5 cerebral cortex and increased neuronal differentiation (Jung et al., 2008). In line with this study, VPA exposure to E12.5 mouse embryos inhibited cell proliferation by 7 days and reduced cell density in the prefrontal cortex at 8 weeks (Kataoka et al., 2013). We have demonstrated that 400 mg/kg of VPA exposure to P14 mice significantly inhibited neurogenesis in the dentate gyrus (Cai et al., 2019). One recent study also reported that VPA exposure during neural tube closure in non-human primates disrupted neurogenesis and reduced Ki67^+^ NPCs and NeuN^+^ mature neurons (Zhao et al., 2019). However, chronic VPA exposure to mice from E1 to birth promoted NPCs proliferation during the early-middle and postnatal neurogenic period (Fujimura et al., 2016). The disparity in cortical neurogenesis might be due to the species, doses, and time of VPA exposure. In this study，*in vitro* model of dorsal forebrain organoid exposure to VPA from day 16 displayed a reduction of the NPCs pool in the higher dose VPA treated organoids similar to those occurring in the first trimester of brain development of ASD subjects with reduced brain size (microcephaly; Jentink et al., 2010; Margulis et al., 2019). In contrast, Cui et al. (2020) showed that VPA exposure to day 11 cortical organoids could enhance the NPCs pool. The inconsistency might be due to the different culture systems of cortical organoids and the time and duration of VPA exposure (Cui et al., 2020). It reported that VPA promoted NPCs apoptosis at therapeutic concentrations (0.3–0.7 mM; Wang et al., 2011a). Here, 250 and 500 μM VPA treatment caused proapoptotic effects on NPCs. These observations suggested that a higher dosage of VPA treatment decreased the NPCs pool due to the inhibition of the proliferation of NPCs and the promotion of its apoptosis. We also noticed that only 500 μM VPA treatment reduced neural differentiation. Species-specific differences occurred in the effects of VPA exposure on brain development appeared in this organoid model.

Cortical organoids are the potential to narrow the gap between cultured cell models and animal studies and recapitulate major architectural characteristics of the fetal brain (Lancaster et al., 2013; Bershteyn et al., 2017). We further detected the distribution of different progenitor cells and deep-layer neurons in 56-day dorsal cortical organoids. We observed that a higher concentration of VPA treatment also dampened the production and migration of IP cells. Typically, 500 μM VPA treatment also reduced early-born neurons. Differential gene analysis further revealed that biological processes related to neurogenesis, generation of neurons, modulation of cell differentiation, embryo development, and positive regulation of cell population proliferation are highly enriched in the DEGs. We further found that a higher concentration of VPA treatment impaired neuronal fate specification of upper layers in the CP of cultured organoids.

The human cerebral cortex is different from that of lower species concerning the expanded oSVZs encompassing abundant populations of oRGs, which generate upper-layer neurons during the development of the human cortex (Fietz et al., 2010; Hansen et al., 2010; Jaffe et al., 2015). Importantly, the radial scaffolds of oRGs guide later-born neuron toward the designated position in the CP, and determine the lamination of CP (Lukaszewicz et al., 2005). Our analyses showed that a well-developed oSVZ with plenty of oRGs is proliferative and actively produces neurons at day 84 of organoids. There was a sharp shrinkage in the thickness of the oSVZ-like structure in the organoids exposed to 250 and 500 μM VPA. Meanwhile, we further confirmed several genes associated with RG and oRG markers significantly decreased in VPA-treated organoids. It seemed that a higher dose of VPA treatment reduced the generation of oRGs and oSVZ-like structures. This impressively explained the phenotypic outcomes in ASD individuals with reduced brain size (microcephaly).

One recent study has found that VPA exposure in human forebrain organoids caused altered many ASD risk gene expression (Meng et al., 2022). Consistent with this, organoid RNA sequencing displayed that VPA treatment changed the levels of ASD risk genes, which overlapped with aberrant genes in ASD brains and patient-derived brain organoids. RNA-seq data also revealed that DEGs were tightly related to the neurodevelopmental dysfunction, such as FBXO32, NANOG, *Wnt8B,* and *GREM1* at an early stage. In particular, the Wnt signaling pathway is linked to neural development in the DEGs. The involvement of Wnt/β-catenin in modulating NPCs proliferation versus differentiation is widely verified (Hirabayashi et al., 2004; Munji et al., 2011). We further confirmed that blocking of β-catenin efficiently rescued NPCs proliferation and neurogenesis defects in 500 μM VPA-treated organoids. Similarly, one study reports that β-catenin overexpression in mice leads to decreased basal NP populations such as IPs (Munji et al., 2011). Given the fact that one of the anticonvulsant effects of VPA is inhibition of HDAC (Phiel et al., 2001; Barrett et al., 2017), an increased level of acetylated histone was also detected in the cerebral organoid exposure to VPA treatment. Furthermore, part of the role of VPA possibly involves HDAC inhibition since a structural analog of VPA lacking the HDAC inhibition activity did not cause phenotype, the changes observed with VPA.

In summary, we found pronounced defects in neurogenesis in VPA-exposed dorsal forebrain organoids characterized by an inhibition of oRG output. Transcriptomic analysis and small-molecule intervention showed dysfunction of the Wnt/β-catenin pathway and inhibition of HDAC play a critical role in the reduced progenitor proliferation and oRG output. The cerebral organoid models provide a new avenue to study the pathophysiology of VPA-induced early developmental deficits in humans.

## Data availability statement

The datasets presented in this study can be found in online repositories. The names of the repository/repositories and accession number(s) can be found in the article/Supplementary material.

## Author contributions

ZZ: conceptualization, methodology, validation, formal analysis, investigation, writing–original draft, and visualization. HYi: software, formal analysis, and data curation. ZD, RX, LY, YC, LW, and DZ: methodology and investigation. XL: software. TL, HG, and JG: investigation. HYa: supervision. MW: project administration. J-AG: project administration and funding acquisition. HX: conceptualization, supervision, and project administration. XF: conceptualization, supervision, project administration, and funding acquisition. All authors read and approved the final manuscript.

## Funding

This study was supported by the National Key R&D Program of China (2021YFA1101203), the National Natural Science Foundation of China (no. 82071544). J-AG was thankful to The Robert A. Welch Foundation for a grant (E-0009).

## Conflict of interest

The authors declare that the research was conducted in the absence of any commercial or financial relationships that could be construed as a potential conflict of interest.

## Publisher’s note

All claims expressed in this article are solely those of the authors and do not necessarily represent those of their affiliated organizations, or those of the publisher, the editors and the reviewers. Any product that may be evaluated in this article, or claim that may be made by its manufacturer, is not guaranteed or endorsed by the publisher.

## Footnotes

^1^ http://www.physics.csbsju.edu/stats/KS-test.n.plot_form.html

^2^https://gene.sfari.org/autdb/HG_Home_do.
