# Supplementary material for: Valproic acid exposure decreases neurogenic potential of outer radial glia in human brain organoids
Source: Front Mol Neurosci. 2022 Nov 29;15:1023765. doi: 10.3389/fnmol.2022.1023765 (PMC9744776; doi:10.3389/fnmol.2022.1023765)
Supplement: Supplementary file 1 [file Data_Sheet_1.pdf]

Supplementary Information for

Valproic acid exposure decreases neurogenic potential of outer radial glia in human brain organoids

Zhenle Zang[1,2], Huachun Yin[2], Zhulin Du[1], Ruxin Xie[1], Ling Yang[1], Yun Cai[1], Liuyongwei Wang[1], Dandan Zhang[1], Xin Li[1], Tianyao Liu[1], Hong Gong[1], Junwei Gao[1], Hui Yang[2], Margaret Warner [3], Jan-Ake Gustafsson[3,4,*], Haiwei Xu[5,*], Xiaotang Fan[1,*]

[1]Department of Developmental Neuropsychology, School of Psychology, Third Military Medical University (Army Medical University), 400038 Chongqing, China.

[2]Department of Neurosurgery, Xinqiao Hospital, Third Military Medical University (Army Medical University), 400037 Chongqing, China.

[3]Center for Nuclear Receptors and Cell Signaling, University of Houston, Houston, TX 77054; [4]Center for Innovative Medicine, Department of Biosciences and Nutrition, Karolinska Institute, 141 86 Novum, Sweden.

[5]Southwest Hospital/Southwest Eye Hospital, Third Military Medical University (Army Medical University), Chongqing, PR China

[*]**Correspondence:**

Corresponding Author

jgustafsson@uh.edu; haiweixu2001@163.com or fanxiaotang2005@163.com

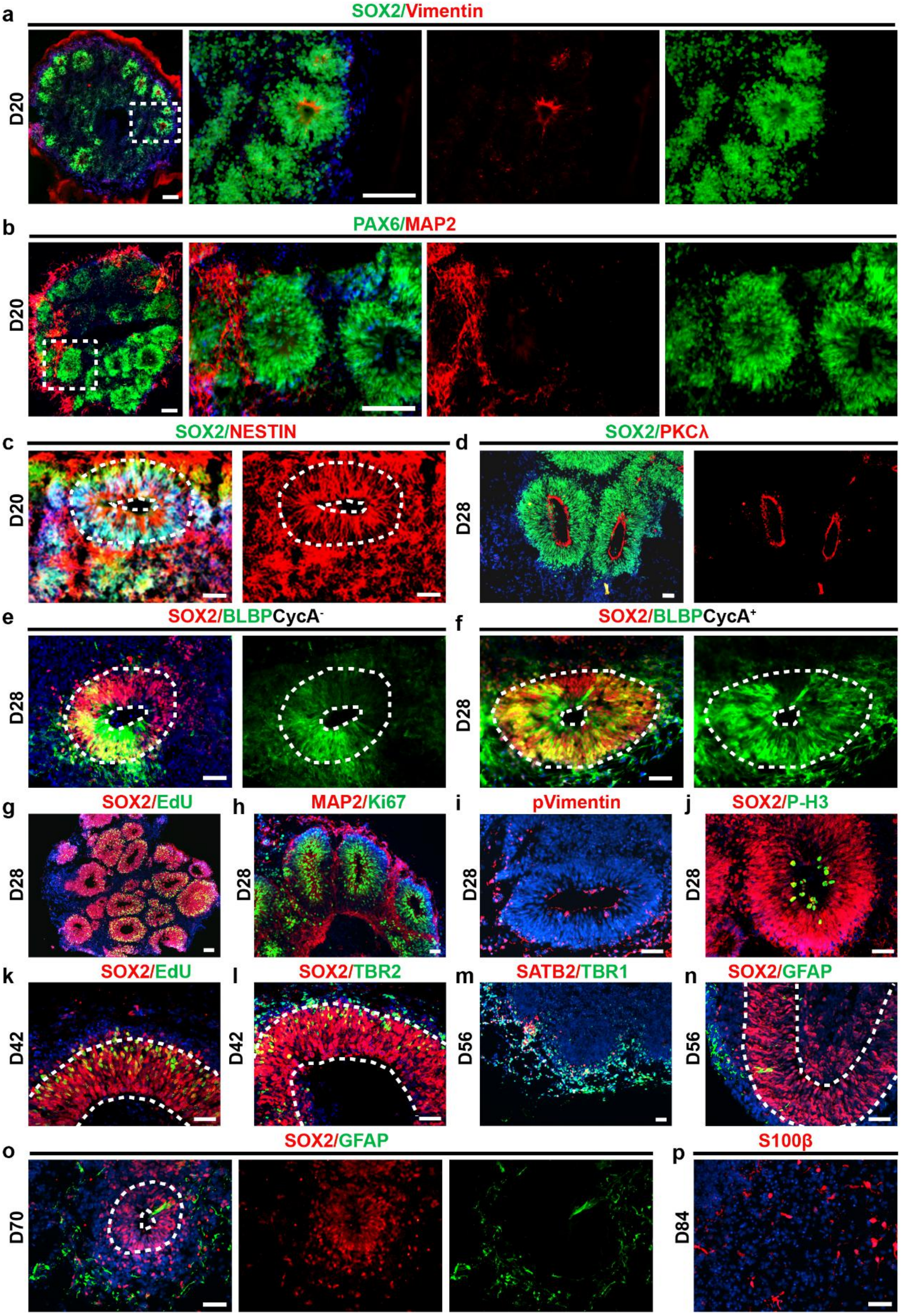

Figure S1. Characterization of hESCs-derived human dorsal forebrain organoids.

a Representative images of dorsal^{CycA} organoids for the progenitor marker SOX2

(green) and Vimentin (red) at day 20. Scale bars,100μm. **b** Representative images of dorsal^{CycA} organoids for PAX6 (green) and neuronal marker MAP2(red) at day 20. Scale bars, 100μm. **c** Representative images of dorsal^{CycA} organoids for SOX2 (Green) and neural stem cell marker NESTIN (red) at day 20. Scale bars, 50μm. **d** Representative images of dorsal^{CycA} organoids for SOX2 (green) and adherent junction marker PKCλ (red) at day 28. Scale bar, 50μm. **e** Representative images of dorsal^{Unt} organoids for SOX2 (red) and the radial glia marker BLBP (green) at day 28. Scale bar, 50μm. **f** Representative images of dorsal^{CycA} organoids for SOX2 (red) and BLBP (green) at day 28. Scale bar, 50μm. **g** Representative images of dorsal^{CycA} organoids for SOX2(red) and EdU (green) after 2-h EdU labeling at day 28. Scale bar, 100μm. **h** Representative images of dorsal^{CycA} organoids for MAP2 (red) and Ki67 (green) at day 28. Scale bar, 50μm.**i** Representative images of dorsal^{CycA} organoids for mitotic cells expressing p-Vimentin (red) at day 28. Scale bar,50μm. **j** Representative images of dorsal^{CycA} organoids for SOX2 (red) and mitotic marker P-H3 (green) at day 28. Scale bar,50μm. **k** Representative images of dorsal^{CycA} organoids for SOX2 (red) and EdU (green) after 2-h EdU labeling at day 42. Scale bar, 50μm. **l** Representative images of dorsal^{CycA} organoids for SOX2 (red) and intermediate progenitor marker TBR2 (green) at day 42. Scale bar, 50μm. **m** Representative images of dorsal^{CycA} organoids for upper-layer neuron marker SATB2 (red) and deep-layer neuron marker TBR1 (green) at day 56. Scale bar, 50μm. **n** Representative images of dorsal^{CycA} organoids for SOX2 (red) and astrocyte marker GFAP (green) at day 56. Scale bar,50μm. **o** Representative images of dorsal^{CycA} organoids for SOX2 (red) and GFAP

(green) at day 70. Scale bar, 50µm. **p** Representative images of dorsal^{CycA} organoids for astrocyte marker S100β at day 84. Scale bar, 50µm.

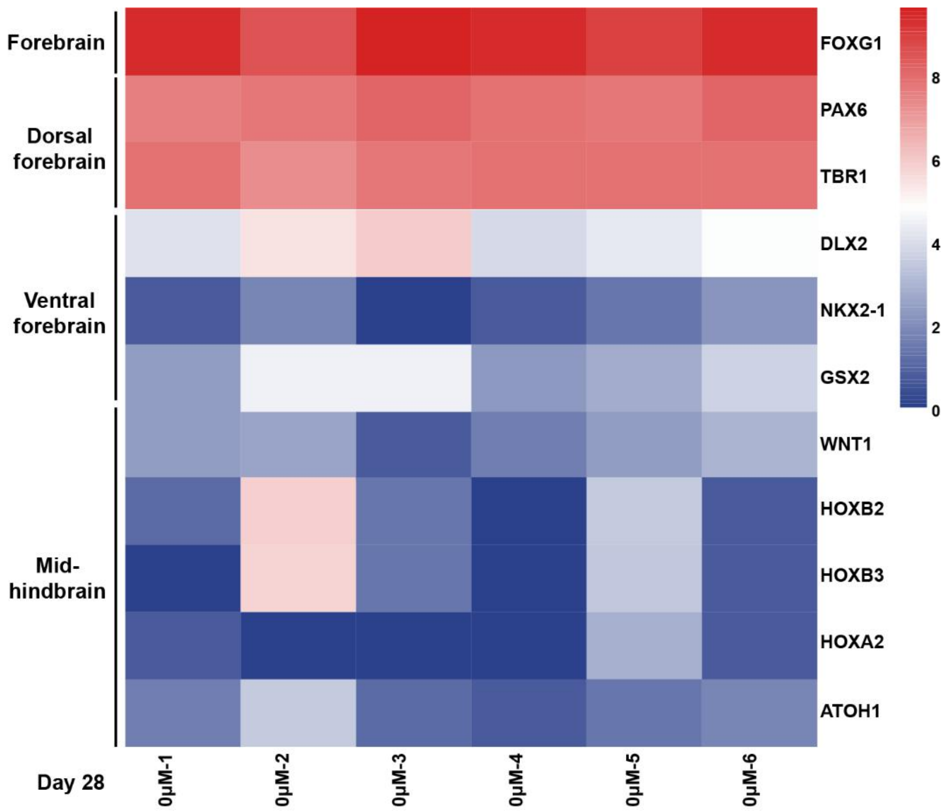

Figure S2. The heatmap showing the mRNA expression levels for forebrain (FOXG1), dorsal (TBR1, PAX6), or ventral (DLX2, NKX2-1, GSX2), or mid-hindbrain (WNT1, ATOH1, HOXB2, HOXB3, or HOXA2) markers in day 28 human dorsal forebrain organoids (n=6 organoids).

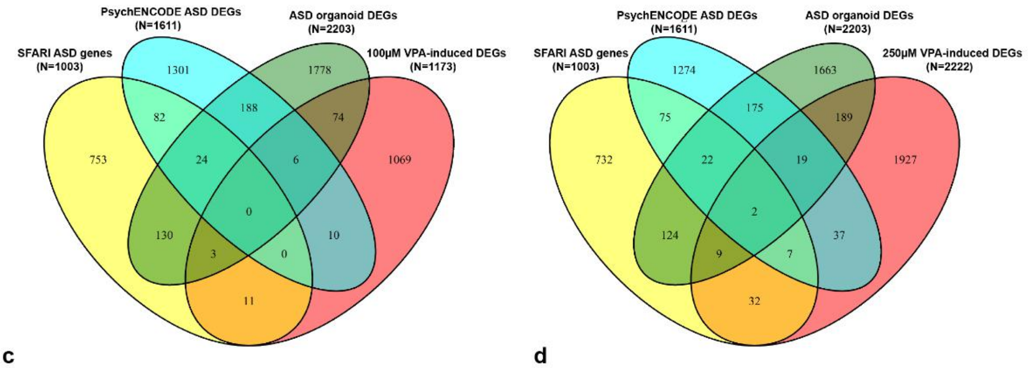

c

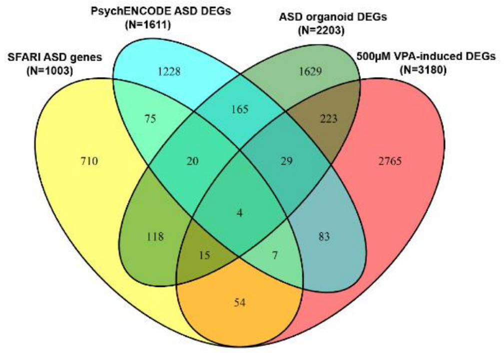

d

Gene	250μM DGEs		500μM DGEs	
	LogFC	FDR	LogFC	FDR
GABRB2	2.33	2.48E-30	2.03	1.12E-78
SCN1A	2.13	1.40E-23	2.06	1.64E-55
GRID2			2.21	6.09E-17
SLC24A2			-3.13	1.43E-25

Figure S3. Enrichment of VPA-induced DEGs with ASD-related risk genes sets.

a, b, c Venn Diagram represents the overlap between 100μM， 250μM and 500μM VPA caused DEGs and three sets (PsychENCODE postmortem brain transcriptome, organoids from four ASD patients transcriptome and the SFARI database) of ASD-related risk genes, respectively. **d** Common DEGs shared in all the gene sets are shown.

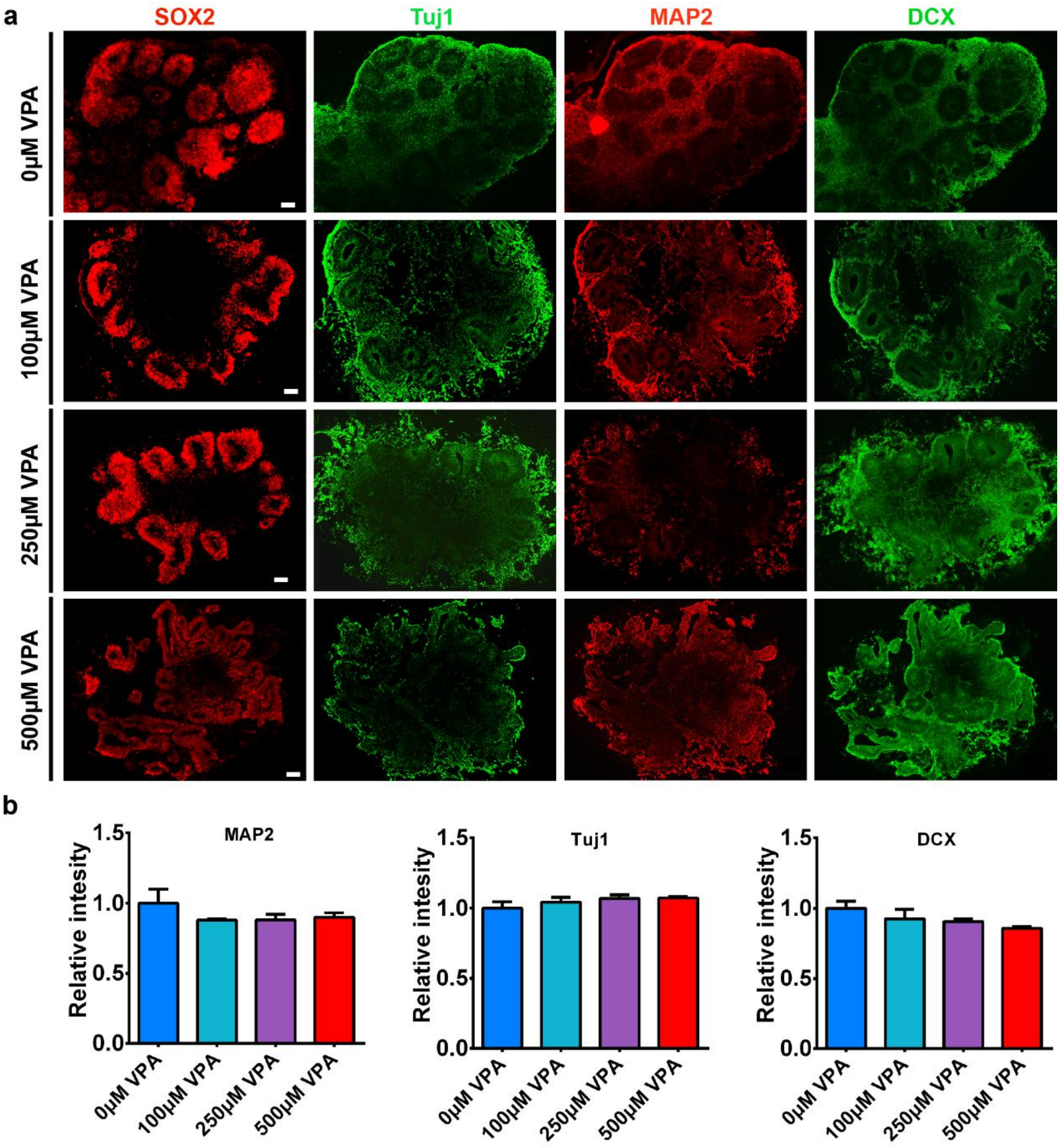

Figure S4. Representative images of day 28 organoid sections stained with antibodies against SOX2 (red), TuJ1 (green), MAP2 (red), and DCX (green).

a Representative images of 0μM, 100μM,250μM, and 500μM VPA treated human dorsal forebrain organoids for SOX2 (red), MAP2 (red), TuJ1(green) and DCX (green) at day 28. Scale bars, 100μm. **b** Quantification of relative intensities of MAP2, TuJ1 and DCX (n=3 organoids each).

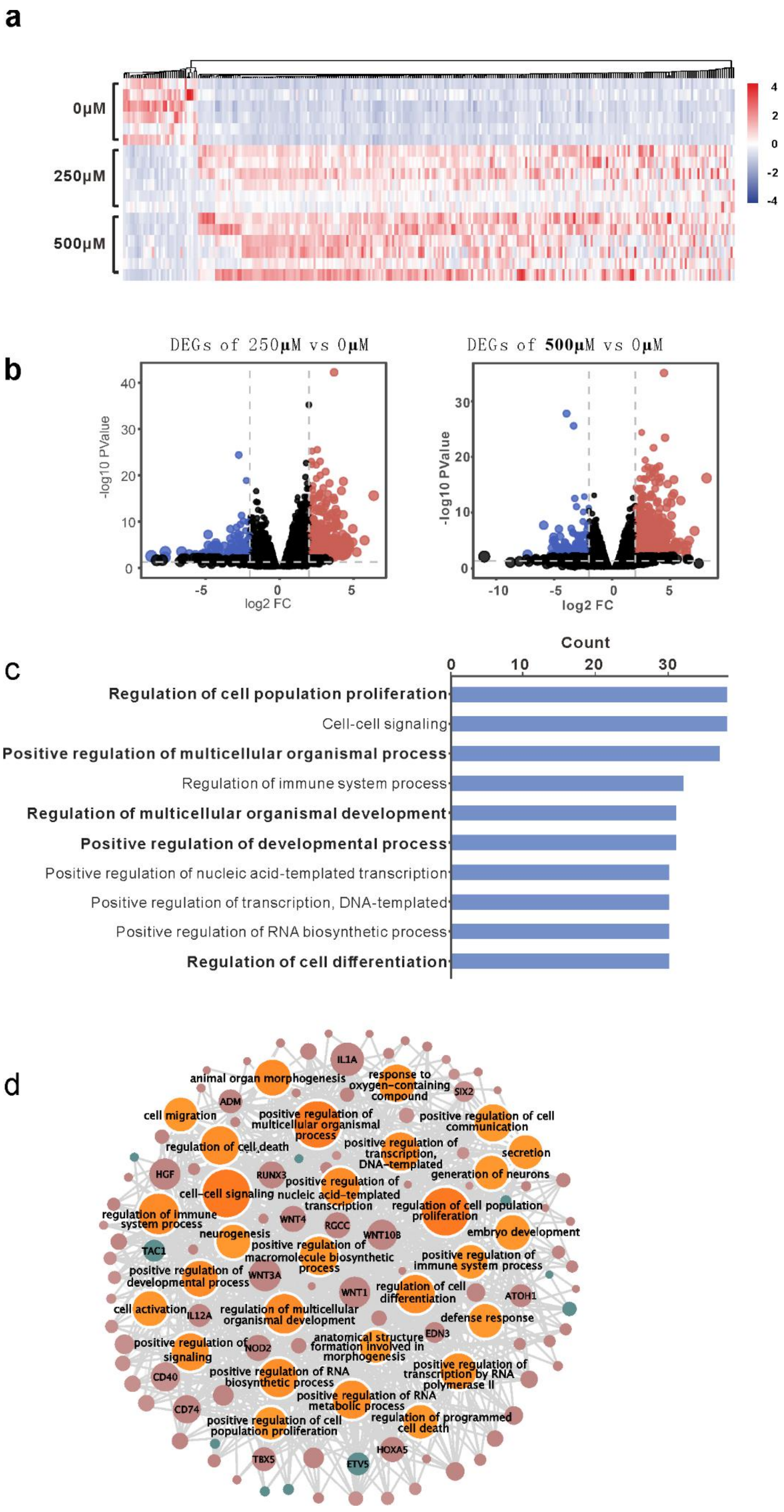
a
0μM
250μM
500μM
4
2
0
-2
-4
b
DEGs of 250μM vs 0μM
DEGs of 500μM vs 0μM
-log10 PValue
log2 FC
c
Count
0
10
20
30
Regulation of cell population proliferation
Cell-cell signaling
Positive regulation of multicellular organismal process
Regulation of immune system process
Regulation of multicellular organismal development
Positive regulation of developmental process
Positive regulation of nucleic acid-templated transcription
Positive regulation of transcription, DNA-templated
Positive regulation of RNA biosynthetic process
Regulation of cell differentiation
d
IL1A
animal organ morphogenesis
response to oxygen-containing compound
SIX2
ADM
cell migration
positive regulation of multicellular organismal process
positive regulation of cell communication
regulation of cell death
positive regulation of transcription, DNA-templated
secretion
HGF
RUNX3
positive regulation of nucleic acid-templated transcription
generation of neurons
cell-cell signaling
regulation of cell population proliferation
regulation of immune system process
WNT4
RGCC
embryo development
neurogenesis
WNT10B
TAC1
positive regulation of macromolecule biosynthetic process
positive regulation of immune system process
positive regulation of developmental process
WNT3A
WNT1
regulation of cell differentiation
ATOH1
cell activation
IL12A
regulation of multicellular organismal development
defense response
positive regulation of signaling
NOD2
anatomical structure formation involved in morphogenesis
EDN3
CD40
positive regulation of RNA biosynthetic process
positive regulation of transcription by RNA polymerase II
positive regulation of RNA metabolic process
CD74
positive regulation of cell population proliferation
regulation of programmed cell death
TBX5
ETV5
HOXA5

Figure S5. The heatmap of expression, GO enrichment analysis for the 280 genes were co-regulated by 250μM and 500μM VPA treatment at day 28.

a The heatmap of expression measures for the 280 genes found DEGs. **b** Volcano plot of DEGs of 250μM and 500μM treated organoids at day 28. Blue circles indicate down-regulated genes and red circles indicate up-regulated genes. **c** Bar plot ranking of the top 10 biological process based on GO enrichment for the 280 DEGs. **d** Enrichment top 30 GO biological process terms for the 280 DEGs. The red nodes represent up-regulated genes, and green nodes represent down-regulated genes. The orange nodes represent GO term, enrichment significance encoded by the node color gradient, where color intensity represents significance. Node size represents the degree of node.

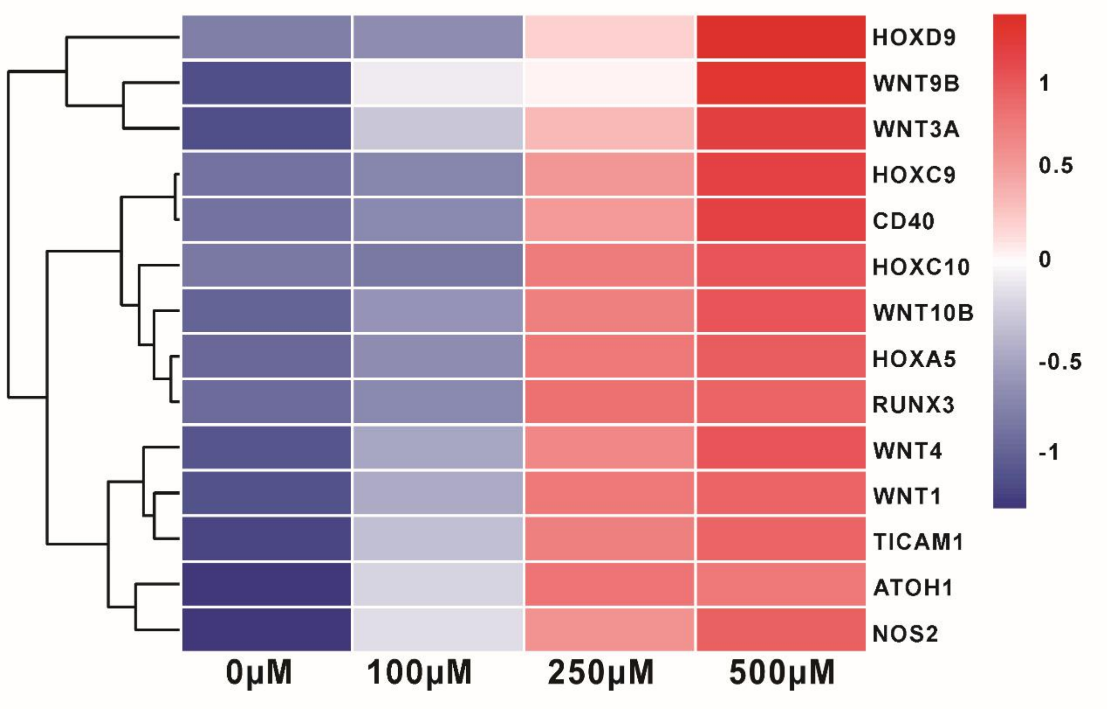

Figure S6. The heatmap showing the mRNA expression levels of the hub gene expression profile in 0μM, 100μM, 250μM, and 500μM VPA treated organoids at day 28.

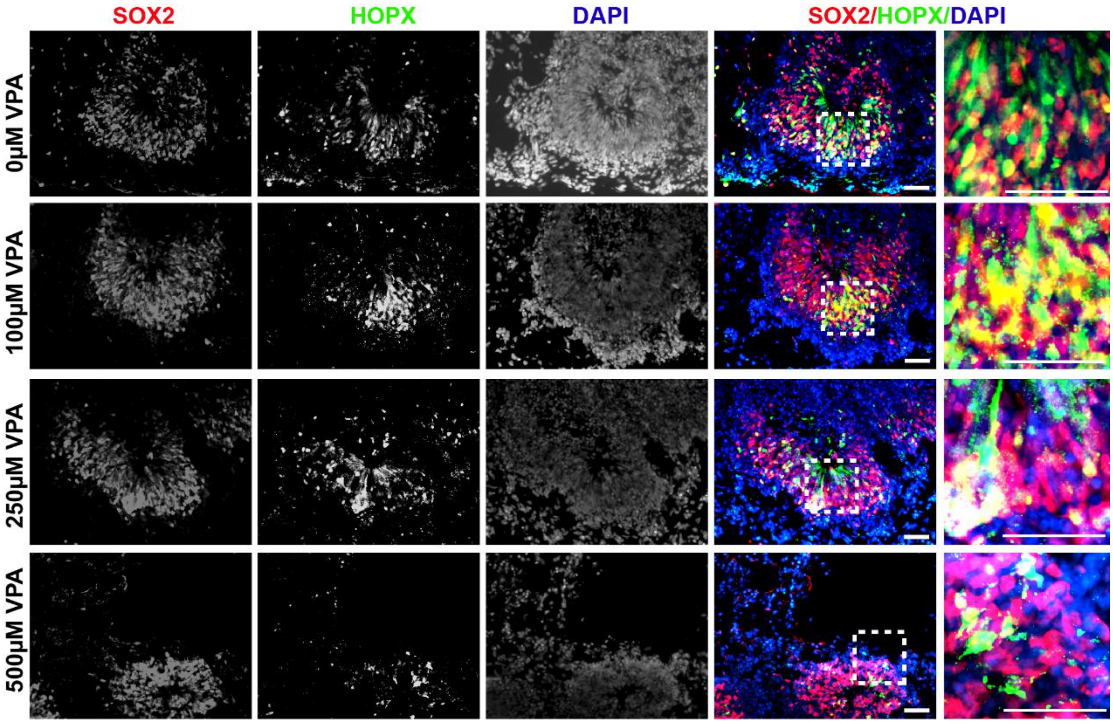

Figure S7. VPA exposure decreased oRG-like cell production in human dorsal forebrain organoids at day 56.

Representative images of 0μM, 100μM, 250μM, and 500μM VPA treated human dorsal forebrain organoids for SOX2 (red) and HOPX (green) staining at day 56. Scale bars, 50μm.

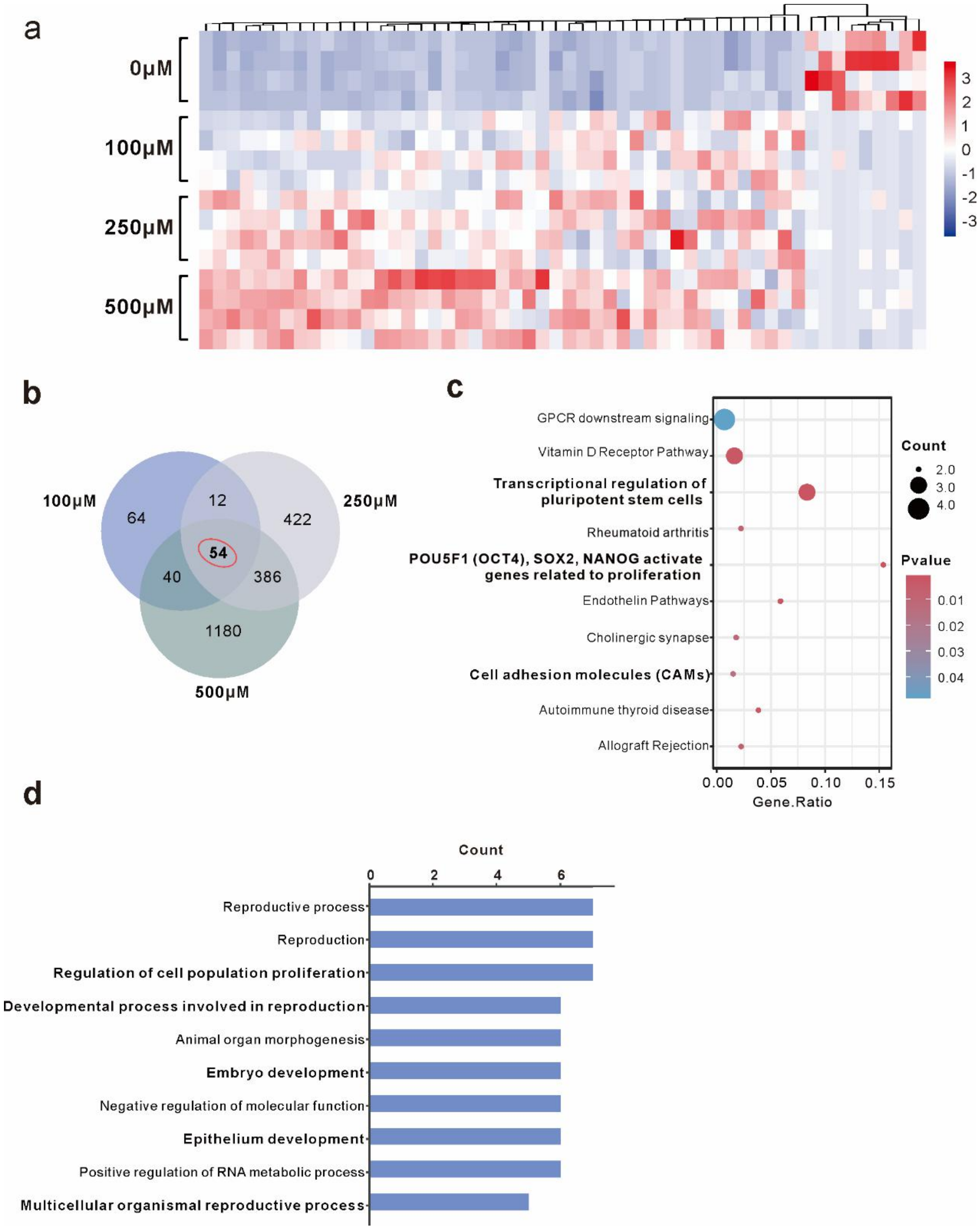

Figure S8. The heatmap of expression, GO enrichment analysis and enriched pathways for the common genes were co-regulated VPA at day 84.

a The heatmap of expression measures for the 54 genes found DEGs. **b** Venn diagram of co-regulated DEGs by VPA. **c** Enriched top ten pathways. **d** Enriched top ten GO biological process terms.

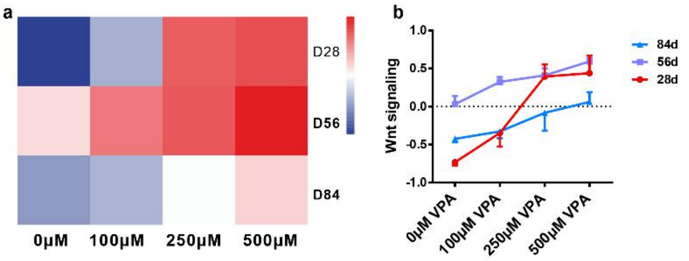

c

Name	Source	FDR
Wnt signaling	Pathway Ontology	0.000098
canonical Wnt signaling	Pathway Ontology	0.000358
Wnt ligand biogenesis and trafficking	REACTOME	0.000358
Wnt signaling pathway	KEGG	0.0114

Figure S9. Wnt signaling pathway was activated in VPA-exposured human dorsal forebrain organoids.

a The heatmap of wnt signaling pathway in 0μM, 100μM, 250μM, and 500μM VPA treated human dorsal forebrain organoids at day 28, 56 and 84. **b** The line graph shows activity scores of Wnt signaling pathway in 0μM, 100μM, 250μM, and 500μM VPA treated human dorsal forebrain organoids at day 28, 56 and 84. for duplicate samples (using R package "GSVA"). **c** Crosstalk pathway of Wnt signaling pathway at day 28. The enriched pathway from KEGG, REACTOME and Pathway Ontology databases.

Table S1. Primary antibodies used in this study

Species	Antigen	Company	Catalog no.	Dilution
Rabbit	FOXG1	Abcam	ab18259	1:300
Rabbit	PAX6	Covance	PRB-278P	1:300
Mouse	SOX2	Abcam	ab79351	1:300
Mouse	Ki67	BD	550609	1:500
Rabbit	DCX	CST	4604	1:300
Rabbit	TBR2	Abcam	ab23345	1:400
Rabbit	SOX2	Abcam	ab97959	1:500
Rabbit	TBR1	Abcam	ab31940	1:400
Rat	CTIP2	Abcam	ab18465	1:100
Mouse	SATB2	Abcam	ab51502	1:100
Rabbit	HOPX	Sigma	HPA030180	1:300
Rabbit	Histone H3 acetyl-K27	Abcam	ab4729	1:1000
Rabbit	Vimentin	Abcam	ab45939	1:500
Mouse	MAP2	Sigma	M1406	1:500
Mouse	NESTIN	Abcam	ab22035	1:400
Rabbit	BLBP	Millipore	ABN14	1:400
Mouse	PKCλ	BD	610207	1:400
Rabbit	Phospho-HistoneH3	CST	9701	1:500,
Mouse	Phospho-Vimentin	MBL	D076-3	1:300
Rabbit	TUJ1	Abcam	ab18207	1:800
Rabbit	GFAP	Dako	Z033401-2	1:400
Rabbit	S100β	Abcam	ab52642	1:500
Rabbit	β-catenin	Abcam	ab32572	1:200

Table S2. The sequence of primers used in this study

Gene	Forward primer (5'-3')	Reverse primer (5'-3')
PAX6	TCCACCCGGCAGAAGATTGTA	TGTCTCGGATTTCCCAAGCAA
NKX2-1	GCCGTACCAGGACACCATG	ATGTTCTTGCTCACGTCCCC
FBXO32	GCCTTTGTGCCTACAACTGAA	CTGCCCTTTGTCTGACAGAAT
NANOG	CCCCAGCCTTTACTCTTCCTA	CCAGGTTGAATTGTTCCAGGTC
WNT8B	AAGGCCGAGAGTGCCTAAG	CTGCGCGGCTACAGAAGTA
GREM1	TCATCAACCGCTTCTGTTACG	GGCTGTAGTTCAGGGCAGTT
ETV5	CAGTCAACTTCAAGAGGCTTGG	TGCTCATGGCTACAAGACGAC
HOPX	GAGACCCAGGGTAGTGATTTGA	AAAAGTAATCGAAAGCCAAGCAC
ATP1A2	CACCACCGAAGATCAGTCTGG	CGCTTAGACACGGAGATGTTC
TNC	TCCCAGTGTTCGGTGGATCT	TTGATGCGATGTGTGAAGACA
WNT1	CGATGGTGGGGTATTGTGAAC	CCGGATTTTGGCGTATCAGAC
WNT9B	TGTGCGGTGACAACCTCAAG	ACAGGAGCCTGATACGCCAT
WNT3A	AGCTACCCGATCTGGTGGTC	CAAACTCGATGTCCTCGCTAC
WNT10B	GTGAGCGAGACCCCACTATG	CACTCTGTAACCTTGCACTCATC
GAPDH	GAAGGTGAAGGTCGGAGTC	GAAGATGGTGATGGGATTTC
